# Design, Synthesis, Phloem Mobility, and Bioactivities of a Series of Phenazine-1-Carboxylic Acid-Amino Acid Conjugates

**DOI:** 10.3390/molecules23092139

**Published:** 2018-08-25

**Authors:** Linhua Yu, Di Huang, Xiang Zhu, Min Zhang, Zongli Yao, Qinglai Wu, Zhihong Xu, Junkai Li

**Affiliations:** 1 College of Agriculture, Yangtze University, Jingmi Road 88, Jingzhou 434025, China; linhuayu531@sina.com (L.Y.); hdhdhd1110@163.com (D.H.); cjdxnxyzx@sina.com (X.Z.); zmzm1017@163.com (M.Z.); y806032526@163.com (Z.Y.); 2 Institute of Pesticides, Yangtze University, Jingmi Road 88, Jingzhou 434025, China; wql106@163.com

**Keywords:** synthesis, phenazine-1-carboxylic acid (PCA), amino acid, phloem mobility, bioactivities

## Abstract

Developing fungicides with phloem mobility that can be applied to leaves to control root or vascular pathogens has long been desirable. To achieve this goal, an efficient and economical strategy involves introducing an amino acid into the existing highly active parent pesticide molecule. Hence, 12 L-phenazine-1-carboxylic acid (PCA)-amino acid conjugates **4a**–**l** were designed and synthesized via a simple synthetic route. In vitro bioassays results showed that all synthesized compounds **4a**–**l** exhibited certain fungicidal activities against six tested fungi. Compound **4c** exhibited relatively good fungicidal activity against *Rhizoctonia solani*, and the EC_50_ value was 0.084 ± 0.006 mmol/L. The phloem mobility experiments revealed that introducing an amino acid to PCA could effectively endow PCA with phloem mobility in *R. communis* L. Among them, nine conjugates were found in phloem sap, and L-PCA-Valine **4d** exhibited the highest phloem mobility. Analysis results from the prediction of the Kleier model indicated that an active carrier-mediated mechanism may be involved in L-PCA-amino acid conjugates—a result that needs to be confirmed and complemented with further tests. The current research provides useful data for modifying non-phloem-mobile fungicidal molecules to phloem-mobile types.

## 1. Introduction

Developing pesticides with phloem mobility has attracted increasing attention in recent years [1,2,3,4,5]. The phloem-mobile pesticides are more economical and efficient because they can be applied to foliage and distributed along with plant growth substances to the site of pathogen infection or damage to control root or vascular diseases of crops [6]. Many structural modification strategies have been used in developing phloem-mobile pesticides [7,8,9,10]. One efficient and economical strategy is to introduce plant growth substrate, such as amino acids or monosaccharides, to the existing highly active parent pesticide molecule [9,10]. For instance, several monosaccharide-fipronil conjugates and their phloem mobility, evaluated in the castor bean system, were reported by Xu et al., and these conjugates exhibited moderate phloem mobility [3]. However, the biological activities of these conjugates almost disappeared. Soon after, the group designed and synthesized a glycinergic-fipronil (GlyF) conjugate by adding an amino acid moiety to fipronil. Linking amino acid can change fipronil into a phloem systemic-type and the uptake process of GlyF was demonstrated to be involved in an active carrier system [11].

In our previous research, in order to endow non-phloem-mobile phenazine-1-carboxylic acid (PCA) with phloem mobility and find higher fungicidal PCA derivatives, PCA was conjugated with 17 amino acid esters [12]. Unfortunately, none of these conjugates exhibited phloem mobility, although they showed excellent fungicidal activities against *Rhizoctonia solani*. After this, we chose to hydrolyze one of them and obtained a novel amino acid conjugate of the fungicide phenazine-1-carboxylic acid, L-2-[(phenazine-1-carbonyl) amino] propanoic acid (L-PA). L-PA has good phloem mobility [13]. The results confirmed that conjugating L-alanine to PCA could endow PCA with phloem mobility in the castor bean system. To the best of our knowledge, the types and contents of amino acids are different in plants [14]. Based on previous research results, we speculate that phloem-mobile compounds could also be developed by conjugating PCA with other L-amino acids. Their phloem transport ability is also different in the castor bean system. To verify our hypothesis, in our current research, 12 PCA-amino acid conjugates were designed and synthesized. Additionally, we report their synthesis, fungicidal activities, and phloem mobility.

## 2. Materials and Methods

### 2.1. Chemicals

Both chemicals and anhydrous solvents of analytical grade were purchased from commercial suppliers. Six phytopathogenic fungi were provided by Institute of Pesticides, Yangtze University, and weed seeds were obtained from Jingzhou, Hubei province, China. The melting points of conjugates **4a**–**l** were determined on an XT-4 melting point apparatus (Shanghai Jingke Industrial Co. Ltd., Shanghai, China). Thin-layer chromatography (TLC) was performed on silica gel 60 F254 (Qingdao Marine Chemical Ltd., Qingdao, China). Column chromatography purification was carried out on silica gel (200–300 mesh) (Qingdao Marine Chemical Ltd., Qingdao, China). Nuclear mass resonance (NMR) spectra were recorded in dimethyl sulfoxide (DMSO)-*d*_6_ solution on a AVANCE III HD 400 NMR spectrometer (Bruker Corporation, Basel, Switzerland).

### 2.2. Plant Materials

Castor bean seeds (*Ricinus communis* L.) were purchased from the Zibo Academy of Agricultural Sciences, Zibo, China. The cultivation of castor seedlings was described previously [2,3]. Then, seedlings 6 days old and average size were selected for the next experiments.

### 2.3. Test Fungus

All six tested phytopathogenic fungi, *Rhizoctonia solani*, *Fusarium graminearum*, *Altemaria solani*, *Fusarium oxysporun*, *Phytophthora capsica*, and *Pyricularia oryzae* were provided by the Institute of Pesticide Research, Yangtze University.

### 2.4. General Synthesis Procedure for Title Compounds ***4a**–**l***

**Scheme 1:**
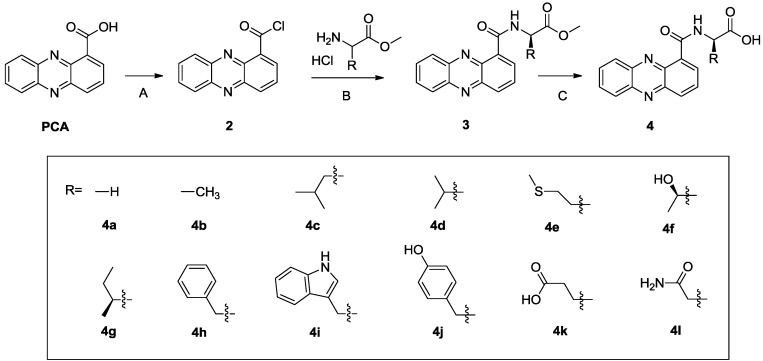
Synthetic route of title compounds **4a**–**l**. Reagents and conditions: (**A**) oxalyl chloride (1.5 equiv), DMF, CH_2_Cl_2_, reflux, 12 h; (**B**) triethylamine (5 equiv), CH_2_Cl_2_, room temperature, 10 h; and (**C**) lithium hydroxide, THF/H_2_O (*v*/*v* = 2:1), room temperature, 10 h.

The PCA-amino acid conjugates **4a**–**l** were synthesized as previously described [12,15]. The synthetic route is described in Scheme 1.

#### 2.4.1. Synthesis of Phenazine-1-Carbonyl Chloride 2

Pure phenazine-1-carboxylic acid (10 mmol) was completely dissolved in 45 mL of dry CH_2_Cl_2_ with 0.1 mmol DMF, cooled at 0–5 °C in an ice water bath and stirred. Then, a solution of oxalyl chloride (15 mmol) in 15 mL CH_2_Cl_2_ was slowly added. The reaction was stirred at reflux temperature for 12 h. The mixture was evaporated under vacuum, and the residue is dissolved in 15 ml anhydrous CH_2_Cl_2_, which was used in the next step without purification [15].

#### 2.4.2. General Procedure for PCA-Amino Acid Ester Conjugates **3a**–**l**

Phenazine-1-carbonyl chloride 2 (10 mmol) completely dissolved in 15 mL of anhydrous CH_2_Cl_2_ was added dropwise to a solution of L-glycine methyl ester hydrochloride (10 mmol), and excessive triethylamine (50 mmol) was the attaching acid agent in CH_2_Cl_2_. The mixture was stirred at room temperature for about 10 h until the reaction was complete. The reaction solution was quenched with water and extracted with 5% Na_2_CO_3_ aqueous solution, and then organic phase was dried over Na_2_SO_4_, filtered and concentrated in vacuum. Finally, pure conjugate **3a** was purified from the obtained crude extract by recrystallization (polyethylene-ethyl acetate (PE-EtOAc) = 1:1). Conjugates **3b**–**3l** were also synthesized by this method [12].

#### 2.4.3. General Procedure for PCA-Amino Acid Conjugates **4a**–**l**

To a solution of conjugate **3a** (10 mmol) in water (15 mL) and THF (30 mL), lithium hydroxide (LiOH) (0.36 g, 15 mmol) was added dropwise, and the reaction mixture was stirred at room temperature for 10 h until the reaction was complete (indicated by TLC). The pH of the reaction mixture was then adjusted to 2 with 1 mol/L HCl and a solid product precipitated was filtered. The THF was removed under vacuum, and the aqueous solution was extracted with 30 mL ethyl acetate three times. The combined ethyl acetate solution (90 mL) was dried over Na_2_SO_4_, filtered and concentrated in a vacuum. The obtained crude extract was purified by column chromatography (PE/EtOAcAcOH, *v*/*v*/*v* = 1:1:0.01–5:1:0.01) to produce pure conjugate **4a**. Conjugates **4b**–**l** were also synthesized by this method [13].

2-(phenazine-1-carboxamido)acetic acid (**4a**): Yellow solid; yield: 76%; m.p. 263–265 °C; ^1^H-NMR (400 MHz, DMSO-*d*_6_) δ 11.21 (t, *J* = 5.2 Hz, 1H, CONH), 8.78 (dd, *J* = 7.2, *J* = 1.6 Hz, 1H, Phenazine-H), 8.46 (m, 2H, Phenazine-H), 8.34–8.27 (m, 1H, Phenazine-H), 8.08 (m, 3H Phenazine-H), 4.23 (d, *J* = 5.2Hz, 2H, CH_2_). ^13^C-NMR (101 MHz, DMSO-*d*_6_) δ 171.85, 164.09, 143.20, 142.92, 141.33, 140.41, 135.01, 133.79, 132.15, 130.74, 129.82, 129.58, 129.54, 129.42, 43.01. HRMS calcd for C_15_H_11_N_3_O_3_ [M + H]^+^: 282.0873, found 282.0868.

(*R*)-2-(phenazine-1-carboxamido)butanoic acid (**4b**): Yellow solid; yield: 76%; m.p. 228–230 °C; ^1^H-NMR (400 MHz, DMSO-*d*_6_) δ 13.04 (s, 1H, COOH), 11.31 (d, *J* = 6.4 Hz, 1H, CONH), 8.73 (dd, *J* = 7.2, *J* = 1.6 Hz, 1H, Phenazine-H), 8.41 (dd, *J* = 8.8, *J* = 1.6 Hz, 1H, Phenazine-H), 8.28 (m, 2H, Phenazine-H), 8.05 (m, 3H, Phenazine-H), 4.66 (m, 1H, CH), 1.60 (d, *J* = 7.2 Hz, 3H, CH_3_). ^13^C-NMR (101 MHz, DMSO-*d*_6_) δ 174.58, 163.73, 143.31, 143.03, 141.29, 140.44, 135.03, 133.90, 132.84, 132.25, 130.83, 129.74, 129.44, 129.32, 43.18, 18.58. HRMS calcd for C_16_H_13_N_3_O_3_ [M + H]^+^: 296.1030, found 296.1020. 

(*R*)-4-methyl-2-(phenazine-4-carboxamido)pentanoic acid (**4c**): Yellow solid; yield: 75%; m.p. 219–221 °C; ^1^H-NMR (400 MHz, DMSO-*d*_6_) δ 12.93 (s, 1H, COOH), 11.03 (d, *J* = 7.2 Hz, 1H, CONH), 8.72 (dd, *J* = 7.2, *J* = 1.6 Hz, 1H, Phenazine-H), 8.45 (dd, *J* = 8.8, *J* = 1.6 Hz, 1H, Phenazine-H), 8.35–8.21 (m, 2H, Phenazine-H), 8.08 (m, 3H, Phenazine-H), 4.69 (m, 1H, acylamino-CH), 1.92 (m, 2H, CH_2_), 1.82 (m, 1H, methyl-CH-methyl), 1.02 (dd, *J* = 11.2, *J* = 6.0 Hz, 6H, methenyl-(CH_3_)_2_). ^13^C-NMR (101 MHz, DMSO-*d*_6_) δ 174.41, 164.17, 143.32, 143.05, 141.39, 140.50, 134.88, 133.77, 132.93, 132.22, 130.84, 129.88, 129.80, 129.17, 51.97, 41.21, 25.30, 23.33, 22.38. HRMS calcd for C_19_H_20_N_3_O_3_ [M + H]^+^: 338.1499, found 338.1492.

(*R*)-3-methyl-2-(phenazine-4-carboxamido)butanoic acid (**4d**): Yellow solid; yield: 78%; m.p. 246–248 °C; ^1^H-NMR (400 MHz, DMSO-*d*_6_) δ 13.02 (s, 1H, COOH), 11.20 (d, *J* = 8.0 Hz, 1H, CONH), 8.77 (dd, *J* = 7.2, *J* = 1.6 Hz, 1H, Phenazine-H), 8.44 (dd, *J* = 8.8, *J* = 1.6 Hz, 1H, Phenazine-H), 8.29 (dd, *J* = 8.4, *J* = 1.6 Hz, 1H, Phenazine-H), 8.23–8.15 (m, 1H, Phenazine-H), 8.06 (m, 3H, Phenazine-H), 4.66 (dd, *J* = 8.0, *J* = 4.4 Hz, 1H, acylamino-C*H*), 2.41 (m, 1H, methyl-CH-methyl), 1.13 (dd, *J* = 29.4, *J* = 6.8 Hz, 6H, methenyl-(CH_3_)_2_). ^13^C-NMR (101 MHz, DMSO-*d*_6_) δ 173.33, 164.19, 143.33, 142.96, 141.13, 140.45, 135.26, 133.94, 133.05, 132.14, 130.80, 129.79, 129.37, 128.86, 58.45, 30.92, 19.86, 18.51. HRMS calcd for C_18_H_17_N_3_O_3_ [M + H]^+^: 324.1343, found 324.1335.

(*R*)-4-(methylthio)-2-(phenazine-4-carboxamido)butanoic acid (**4e**): Yellow solid; yield: 74%; m.p. 211–213 °C; ^1^H-NMR (400 MHz, DMSO-*d*_6_) δ 13.25 (s, 1H, COOH), 11.21 (dd, *J* = 7.2, *J* = 2.4 Hz, 1H, CONH), 8.72 (m, 1H, Phenazine-H), 8.46 (dd, *J* = 8.8, *J* = 1.6 Hz, 1H, Phenazine-H), 8.41–8.35 (m, 1H, Phenazine-H), 8.35–8.28 (m, 1H, Phenazine-H), 8.14–8.02 (m, 3H, Phenazine-H), 4.84 (m, 1H, acylamino-CH), 3.11–2.75 (m, 2H, CH_2_-S), 2.56 (s, 3H, CH_3_), 2.48–2.24 (m, 2H, CH_2_-methylene-S). ^13^C-NMR (101 MHz, DMSO-*d*_6_) δ 173.16, 164.42, 143.25, 143.07, 141.45, 140.45, 134.80, 133.80, 132.80, 132.28, 130.84, 129.89, 129.71, 129.45, 52.55, 49.96, 38.47, 25.27. HRMS calcd for C_18_H_17_N_3_O_3_S [M + H]^+^: 356.1063, found 356.1057.

(2*R*,3*R*)-3-hydroxy-2-(phenazine-4-carboxamido)butanoic acid (**4f**): Yellow solid; yield: 72%; m.p. 227–229 °C; ^1^H-NMR (400 MHz, DMSO-*d*_6_) δ 11.49 (d, *J* = 7.6 Hz, 1H, CONH), 8.81 (dd, *J* = 7.2, 1.5 Hz, 1H, Phenazine-H), 8.47 (dd, *J* = 8.8, *J* = 1.6 Hz, 1H, Phenazine-H), 8.37–8.19 (m, 2H, Phenazine-H), 8.17–7.97 (m, 3H, Phenazine-H), 4.57 (dd, *J* = 7.6, *J* = 2.4 Hz, acylamino-CH), 4.44 (m, 1H, hydroxyl-CH), 1.29 (d, *J* = 6.4 Hz, 3H, CH_3_). ^13^C-NMR (101 MHz, DMSO-*d*_6_) δ 172.65, 164.46, 143.36, 142.99, 141.25, 140.55, 135.28, 133.96, 132.80, 132.27, 130.90, 129.73, 129.40, 129.35, 66.97, 59.16, 21.62. HRMS calcd for C_17_H_15_N_3_O_4_ [M + H]^+^: 326.1135, found 326.1129.

(*2R,3R*)-3-methyl-2-(phenazine-4-carboxamido)pentanoic acid (**4g**): Yellow solid; yield: 75%; m.p. 219–221 °C; ^1^H-NMR (400 MHz, DMSO-*d*_6_) δ 12.98 (s, 1H, COOH), 11.23 (d, *J* = 8.0Hz, 1H, CONH), 8.76 (dd, *J* = 7.2, *J* = 1.6 Hz, 1H, Phenazine-H), 8.44 (dd, *J* = 8.8, *J* = 1.6 Hz, 1H, Phenazine-H), 8.30 (dd, *J* = 8.4, *J* = 1.6 Hz, 1H, Phenazine-H), 8.18 (dd, *J* = 8.8, *J* = 1.6 Hz, 1H, Phenazine-H), 8.13–7.99 (m, 3H, Phenazine-H), 4.70 (dd, *J* = 8.0, *J* = 4.8 Hz, 1H, acylamino-CH), 2.13 (m, 1H, methyl-CH-methylene), 1.81–1.59 (m, 1H, CH_2_), 1.54–1.34 (m, 1H, CH_2_), 1.11–0.90 (m, 6H, CH_3_-methenyl-methylene-CH_3_). ^13^C-NMR (101 MHz, DMSO-*d*_6_) δ 173.26, 164.03, 143.34, 142.98, 141.17, 140.46, 135.24, 133.94, 133.08, 132.17, 130.82, 129.82, 129.36, 128.83, 57.61, 37.64, 25.69, 16.47, 12.15. HRMS calcd for C_19_H_19_N_3_O_3_ [M + H]^+^: 338.1499, found 338.1492.

(*R*)-2-(phenazine-1-carboxamido)-3-phenylpropanoic acid (**4h**): Yellow solid; yield: 78%; m.p. 104–106 °C; ^1^H-NMR (400 MHz, DMSO-*d*_6_) δ 11.18 (d, *J* = 7.2 Hz, 1H, CONH), 8.77 (dd, *J* = 7.2, *J* = 1.6 Hz, 1H, Phenazine-H), 8.45 (dd, J = 8.8, *J* = 1.6 Hz, 1H, Phenazine-H), 8.34–8.25 (m, 1H, Phenazine-H), 8.14–7.98 (m, 4H, Phenazine-H), 7.37–7.29 (m, 2H, Ar-H), 7.26–7.11 (m, 3H, Ar-H), 5.03–4.92 (m, 1H, acylamino-CH), 3.35 (dd, *J* = 6.0, *J* = 3.6 Hz, 2H, Ar-CH_2_). ^13^C-NMR (101 MHz, DMSO-*d*_6_) δ 173.28, 163.83, 143.22, 142.92, 141.17, 140.37, 137.80, 135.16, 133.90, 132.64, 132.18, 130.78, 129.81, 129.62, 129.31, 128.70, 126.97, 54.89, 37.56, 21.56. HRMS calcd for C_22_H_17_N_3_O_3_ [M + H]^+^: 372.1343, found 372.1335.

(*2R*)-3-(3a,7a-dihydro-1H-indol-3-yl)-2-(phenazine-1-carboxamido)-propanoic acid (**4i**): Red solid; yield: 73%; m.p. 194–196 °C; ^1^H-NMR (400 MHz, DMSO-*d*_6_) δ11.23 (d, *J* = 7.2 Hz, 1H, CONH), 10.89 (s, 1H, NH), 8.79 (dd, *J* = 7.2, *J* = 1.2 Hz, 1H, Phenazine-H), 8.44 (dd, *J* = 8.4, *J* = 1.2 Hz, 1H, Phenazine-H), 8.27 (d, *J* = 8.4 Hz, 1H, Phenazine-H), 8.09 (dd, *J* = 8.4, *J* = 7.2 Hz, 1H, Phenazine-H), 8.01 (t, *J* = 7.2 Hz, 1H, Phenazine-H), 7.94 (t, *J* = 7.2 Hz, 1H, Phenazine-H), 7.74 (d, *J* = 8.4 Hz, 1H, Phenazine-H), 7.58 (d, *J* = 8.0 Hz, 1H, Ar-H), 7.31–7.23 (m, 2H, Ar-H), 6.98 (t, *J* = 7.6 Hz, 1H, Ar-H), 6.82 (t, *J* = 7.6 Hz, 1H, C = CH), 5.02 (d, *J* = 6.4 Hz, 1H, acylamino-CH), 3.48 (d, *J* = 5.6 Hz, 2H). ^13^C-NMR (101 MHz, DMSO-*d*_6_) δ 173.78, 163.99, 143.24, 142.91, 141.13, 140.39, 136.58, 135.25, 133.96, 132.60, 132.16, 130.81, 129.58, 129.29, 129.20,128.00, 124.17, 121.41, 118.82, 111.84, 109.84, 54.23, 27.74, 0.65. HRMS calcd for C_24_H_18_N_4_O_3_ [M + H]^+^: 411.1451, found 411.1445.

(*R*)-3-(4-hydroxyphenyl)-2-(phenazine-1-carboxamido)propanoic acid (**4j**): Yellow solid; yield: 76%; m.p. 240–241 °C; ^1^H-NMR (400 MHz, DMSO-*d*_6_) δ 13.01 (s, 1H, COOH), 11.12 (d, *J* = 7.2 Hz, 1H, CONH), 9.22 (s, 1H, Ar-OH), 8.76 (dd, *J* = 7.2, *J* = 1.6 Hz, 1H, Phenazine-H), 8.42 (dd, *J* = 8.8, 1.6 Hz, 1H, Phenazine-H), 8.33–8.18 (m, 1H, Phenazine-H), 8.14–7.92 (m, 4H, Phenazine-H), 7.13 (d, *J* = 8.0 Hz, 2H, Ar-H), 6.62 (d, *J* = 8.0 Hz, 2H, Ar-H), 5.04–4.82 (m, 1H, acylamino-CH), 3.23 (d, *J* = 6.0 Hz, 2H, CH_2_). ^13^C-NMR (101 MHz, DMSO-*d*_6_) δ 173.35, 163.91, 156.61, 143.21, 142.92, 141.13, 140.35, 135.24, 133.96, 132.65, 132.15, 130.74, 130.72, 129.64, 129.23, 129.15, 127.50, 115.62, 54.86, 36.74. HRMS calcd for C_22_H_17_N_3_O_4_ [M + H]^+^: 388.1292, found 388.1285.

(*R*)2-(phenazine-4-carboxamido)pentanedioic acid (**4k**): Yellow solid; yield: 75%; m.p. 204–206 °C; ^1^H-NMR (400 MHz, DMSO-*d*_6_) δ 12.62 (s, 2H, COOH), 11.05 (d, *J* = 7.2 Hz, 1H, CONH), 8.69 (dd, *J* = 7.2, *J* = 1.6 Hz, 1H, Phenazine-H), 8.46 (dd, J = 8.8, J = 1.6 Hz, 1H, Phenazine-H), 8.37–8.25 (m, 2H, Phenazine-H), 8.13–8.01 (m, 3H, Phenazine-H), 4.75 (m, 1H, acylamino-CH), 2.58–2.51 (m, 2H, carboxyl-CH_2_), 2.23 (m, 2H, methyne-CH_2_). ^13^C-NMR (101 MHz, DMSO-*d*_6_) δ 174.22, 173.57, 164.33, 143.22, 143.01, 141.39, 140.42, 134.68, 133.69, 132.72, 132.18, 130.79, 130.01, 129.70, 129.33, 52.70, 30.51, 27.45. HRMS calcd for C_18_H_15_N_3_O_5_ [M + H]^+^: 354.1084, found 354.1077.

(*R*)-4-amino-4-oxo-2-(phenazine-4-carboxamido)butanoic acid (**4l**): Yellow solid; yield: 74%; m.p. 224–226 °C; ^1^H-NMR (400 MHz, DMSO-*d6*) δ 12.89 (s, 2H, CONH_2_), 11.67 (d, *J* = 8.0 Hz, 1H, CONH), 8.81 (dd, *J* = 7.2, *J* = 1.6 Hz, 1H, Phenazine-H), 8.49–8.37 (m, 2H, Phenazine-H), 8.34–8.26 (m, 1H, Phenazine-H), 8.16–7.99 (m, 3H, Phenazine-H), 5.04 (m, 1H, acylamino-CH), 3.11 (dd, *J* = 17.2, 4.8 Hz, 1H, CH_2_), 2.95 (dd, *J* = 17.2, 4.8 Hz, 1H, CH_2_). ^13^C-NMR (101 MHz, DMSO-*d*_6_) δ 173.08, 172.85, 163.90, 143.25, 142.97, 141.31, 140.42, 135.41, 134.06, 132.67, 132.23, 130.82, 129.66, 129.39, 129.00, 49.28, 36.80. HRMS calcd for C_17_H_14_N_4_O_4_ [M + H]^+^: 339.1088, found 339.1085.

### 2.5. Assay of Fungicidal Activities 

Twelve title compounds were screened for their in vitro antifungal activities against *Rhizoctonia solani*, *Fusarium graminearum*, *Altemaria solani*, *Fusarium oxysporun*, *Phytophthora capsica*, and *Pyricularia oryzae* by the mycelium growth rate test [15,16].

The primary fungicidal activities experiment was performed in an isolated culture under sterile conditions. All synthesized compounds weighed out and the appropriate amount was dissolved in 1 mL acetone, diluted with a sterile 1% Tween 80 aqueous solution and then added to sterile potato dextrose agar (PDA, 49 mL), and the final concentrations of the tested compounds were 0.2 mmol/L (1 mL sterile water was used as blank assay). Mycelial discs of tested fungi grown on potato dextrose agar medium were taken from the margins of the colony using a 6 mm drill and placed on the center of the PDA containing the tested compound. Then, the petri plates were cultivated at 28 ± 1 °C, and all operations were under a sterile condition. After 72 h, the extended diameter of the circle mycelium was calculated. Acetone and phenazine-1-carboxylic acid in a sterile 1% Tween 80 aqueous solution served as the negative control and positive control, respectively. Every sample had three replicates. All statistical analyses were performed using DPS 7.05 software. The relative inhibition rate of the circle mycelium was calculated as follows: Relative inhibition rate (%) = [(CK – PT)/(CK – 6 mm)] × 100%, where CK represents the colony diameter during the blank assay and PT represents the colony diameter during testing.

### 2.6. Phloem Sap Collection

The method of phloem sap collection was the same as described in previous studies [2,13]. The cotyledons were washed after their endosperm was peeled off. Then, they were immersed in acidic solution (pH 5.5) containing 200 μmol/L of every target compound for 2 h. The roots were cultivated in deionized water containing 0.5 mmol/L CaCl_2_. The hypocotyl was severed at the hook of seedlings according to recently described procedures after 2 h. Phloem sap collected after 2 h was discarded as the test compound was nearly not detected in the first 2 h in phloem sap, as reported by our previous research [13]. Then, phloem sap between 2 and 4 h was collected for detected.

### 2.7. Analytical Methods

The phloem sap was diluted with pure water (phloem sap/pure water, 1:2, *v*/*v*), and was filtered with a 0.22 μm filter before analysis via a LC7000 high-performance liquid chromatography (HPLC) instrument (Hanon Instruments, Jinan, China) and a C18 reversed-phase column (5 μm, 150 × 4.6 mm inner diameter) was used for separations at 25 °C. The mobile phase was made of methanol and water containing 0.1% phosphoric acid under different proportions for different test compounds at a flow rate of 0.8 mL/min, and the injection volume was 10 μL. The HPLC characteristic spectra are available in Supplementary Information.

## 3. Results and Discussion

### 3.1. Synthesis

Scheme 1 outlines the three-step synthetic route for PCA-amino acid conjugates. Treatment of PCA with oxalyl chloride at reflux temperature in CH_2_Cl_2_ solution afforded intermediate **2** after the evaporation of CH_2_Cl_2_ [15]. Intermediate **2** was treated with the corresponding L-amino acid methyl ester hydrochloride by using excessive triethylamine (5 equiv) as base at 0 °C to produce the conjugates **3a**–**l** [12]. Hydrolysis of the conjugates **3a**–**l** with LiOH produced the target conjugates **4a**–**l** [13]. The structures of title compounds **4a**–**l** were characterised by proton NMR (^1^H-NMR), carbon NMR (^13^C-NMR), and high resolution mass spectrometry (HR-MS). The ^1^H-NMR, ^13^C-NMR, and HR-MS spectra of compounds **4a**–**l** are available in Supplementary Information.

### 3.2. Fungicidal Activities

Inhibitory rates of title compounds **4a**–**l** against six phytopathogenic fungi are listed in Table 1. Mycelium growth inhibition assay was utilized [17] with fungicide PCA itself as the positive control to evaluate the fungicidal activities of the 12 L-PCA -amino acid conjugates against *Rhizoctonia solani*, *Fusaium graminearum*, *Altemaria solani*, *Fusarium oxysporum*, *Phytophthora capsica*, and *Pyricularia oryzae* at a concentration of 0.2 mmol/L. Among the synthesized compounds, all the test compounds had certain in vitro fungicidal activities, but most lower than PCA. Among them, the compound **4c** exhibited relatively good fungicidal activity against *R. solani* compared to the positive control PCA at the test concentrations, with the EC_50_ being 0.084 ± 0.006 mmol/L and 0.080 ± 0.008 mmol/L, respectively (Table 2).

### 3.3. Phloem Mobility in R. communis Seedlings

Phloem mobility of PCA-amino acid conjugates **4a**–**l** was evaluated using the castor bean system, which is an ideal biological model widely employed to study the phloem mobility of xenobiotics given their thin and highly permeable cuticles [5,18]. The phloem sap was collected when the cotyledons were incubated with each title compound of 200 μmol/L for two hours. The composition of phloem sap with conjugates **4a**−**l** treatments was measured by HPLC.

The detection results of phloem sap are shown in Table 3. As can be seen from the results, nine conjugates were found in phloem sap. This verified our hypothesis that conjugating PCA with other L-amino acids is feasible for endowing fungicidal PCA with phloem mobility. Among these conjugates, L-PCA-Valine (**4d**; 23.94 ± 2.84 µmol/L) showed the best phloem mobility, whereas L-PCA-Tryptophan (**4i**), L-PCA-Tyrosine (**4j**), and L-PCA-Glutamic acid (**4k**) were not detected in phloem sap, thereby indicating that these three conjugates had no phloem mobility. Furthermore, the results also indicate that the phloem mobility of these L-PCA-amino acid conjugates varies significantly. That is to say, the phloem transport ability of these conjugates is different in the current castor bean system.

**Figure 1:**
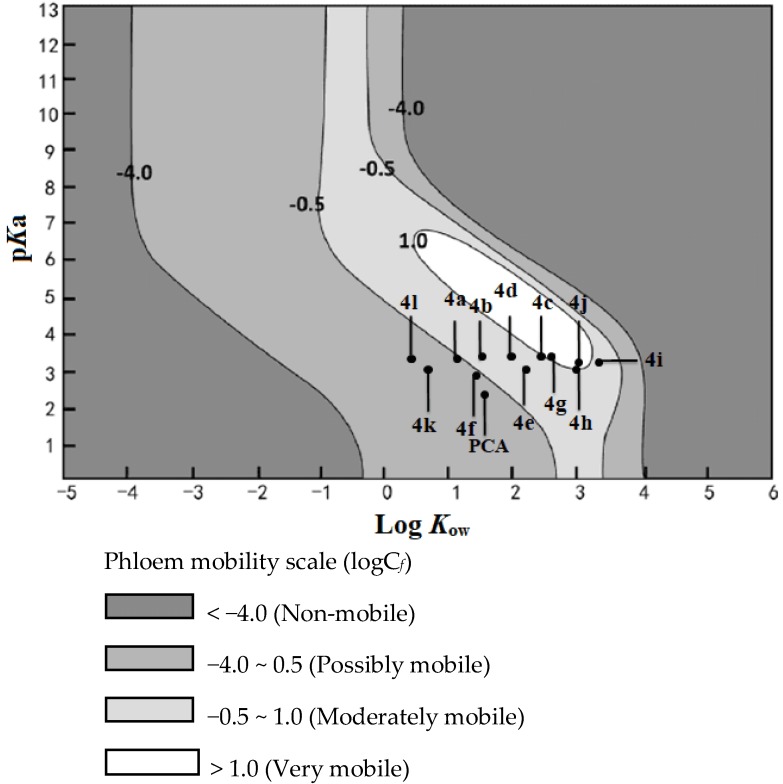
Prediction of phloem mobility of compounds **4a**–**l** and PCA using the Kleier map (logC*_f_* as a function of p*K*a and Log*K*_ow_).

The Kleier model is widely employed to predict whether endogenous compounds or xenobiotics are responsible for the phloem transport ability based on their physical and chemical properties (log *K*_ow_ and p*K*a, respectively) [19,20,21,22]. From a large amount of experimental data reported in the literature [19,20,21,22,23,24], most of the tested xenobiotics, except for some carrier-mediated xenobiotic transport in plants, fit well into the Kleier model. Thus, the physical and chemical properties of tested conjugates **4a**–**l** and PCA are listed in Table 4. Based on the log *K*_ow_ and p*K*a of the conjugates **4a**–**l** and the Kleier prediction model, the prediction of their phloem mobility ranged into the corresponding areas. From the Figure 1, we found that conjugates **4a**, **4f**, **4k**, **4l,** and PCA should be mobile moderately mobile, but these five conjugates, except for **4k** and PCA, exhibited phloem mobility in terms of the experimental results. Additionally, Figure 1 shows that conjugates **4b**, **4c**, **4d**, **4e**, **4h**, and **4i** are located in areas of moderately mobile compounds. Conjugates **4g** and **4j** were located in the very phloem mobile compound area. However, both **4i** and **4j** were found to be non-phloem mobile using the *Ricinus* system, so therefore they both violate the Kleier model. The phloem mobility of some synthesized molecules with an amino acid or a sugar moiety, such as glycinergic-fipronil (GlyF) [11] and glucose-fipronil conjugate (GTF) [25], were proven to involve active carrier processes that can recognize and load these conjugates into phloem sieve tubes. Therefore, it is likely that an active carrier-mediated mechanism is also involved in phenazine-1-carboxylic acid-amino acid conjugates uptake as well as in the systemic nature of glycinergic-fipronil conjugate and glucose-fipronil conjugate [11,25]. The carrier-mediated mechanisms of these PCA-amino acid conjugates requires further study. 

## 4. Conclusions

In summary, 12 L-PCA-amino acid conjugates **4a**–**l** were designed and synthesized using a simple synthetic route. In vitro bioassays results showed that all synthesized compounds **4a**–**l** exhibited certain fungicidal activities against six tested fungi, but most lower than PCA. Compound **4c** exhibited relatively good fungicidal activity against *R. solani*, and the EC_50_ value was 0.084 ± 0.006 mmol/L. The phloem mobility experiments revealed that introducing an amino acid to PCA could effectively endow PCA with phloem mobility in *R. communis* L. Among them, nine conjugates were found in phloem sap, and L-PCA-Valine **4d** exhibited the highest phloem mobility. Analysis results from the prediction of the Kleier model indicated that an active carrier-mediated mechanism may be involved in L-PCA-amino acid conjugates, which is a result that requires confirmation and further tests. The current research provides useful data for transforming non-phloem-mobile fungicidal molecules to phloem-mobile types.

## Figures and Tables

**Table 1:** *In vitro* fungicidal activities of title compounds against six pathogenic fungi.

Compound	*R. solani*	*F. graminearum*	*A. solani*	*F. oxysporum*	*P. capsici*	*P. oryzae*
**4a**	54.5 ± 1.0	40.7 ± 1.0	16.3 ± 0.0	34.0 ± 1.4	16.8 ± 1.3	34.1 ± 1.1
**4b**	22.7 ± 1.1	34.0 ± 1.4	25.4 ± 0.6	27.9 ± 1.3	30.6 ± 0.8	23.3 ± 0.6
**4c**	74.2 ± 0.4	28.5 ± 1.7	24.6 ± 1.2	44.8 ± 0.6	31.5 ± 0.0	33.5 ± 1.6
**4d**	48.9 ± 1.9	26.8 ± 1.0	26.7 ± 0.5	25.4 ± 0.7	40.9 ± 1.7	35.9 ± 1.1
**4e**	25.5 ± 1.0	28.0 ± 0.6	18.3 ± 0.1	20.4 ± 0.7	18.8 ± 0.7	42.7 ± 0.6
**4f**	35.4 ± 0.0	20.5 ± 1.2	15.6 ± 0.7	24.0 ± 0.0	15.4 ± 0.6	32.2 ± 1.1
**4j**	40.0 ± 1.8	26.8 ± 1.0	33.6 ± 1.8	36.9 ± 0.0	50.3 ± 0.7	38.4 ± 0.5
**4h**	24.0 ± 1.4	26.2 ± 0.6	18.3 ± 1.2	46.9 ± 1.4	47.6 ± 1.2	31.6 ± 0.2
**4i**	33.3 ± 0.9	35.5 ± 0.2	36.4 ± 0.7	29.0 ± 1.4	32.9 ± 1.8	37.2 ± 0.6
**4j**	37.4 ± 1.4	25.7 ± 0.6	18.3 ± 1.2	21.1 ± 0.7	21.5 ± 0.0	37.8 ± 1.1
**4k**	25.0 ± 0.5	30.9 ± 0.6	14.2 ± 1.2	21.8 ± 1.2	17.4 ± 1.2	37.2 ± 0.6
**4l**	34.8 ± 1.4	14.1 ± 0.6	16.9 ± 1.8	23.3 ± 1.4	23.5 ± 0.0	39.0 ± 1.6
**PCA**	76.2 ± 0.5	84.5 ± 1.5	73.6 ± 1.4	70.6 ± 1.4	100 ± 0.0	77.8 ± 1.1

Note: Inhibitory ratio (%) was measured at a concentration of 0.2 mmol/L. Each treatment repeated three times (mean ± SD). PCA served as the positive control.

**Table 2:** In vitro fungicidal activities of compounds **4c** and PCA against *R. solani*.

Compound	Regression Equation	Correlation Coefficient (*r^2^*)	EC_50_ ± SD (mmol/L)
**4c**	*y* = 0.7139 *x* + 5.7671	0.9928	0.084 ± 0.006
**PCA**	*y* = 1.5830 *x* + 6.7335	0.9467	0.080 ± 0.008

**Table 3:** Analysis of phloem sap.

Compound	Conjugated Amino Acid	Concentration in Phloem Sap *^a^* (µmol/L)	Significant Difference *^c^*
**4a**	Glycine (Gly)	13.48 ± 1.47	bc
**4b**	L-Alanine (L-Ala)	14.36 ± 1.60	b
**4c**	L-Leucine (L-Leu)	8.21 ± 1.44	de
**4d**	L-Valine (L-Val)	23.94 ± 2.84	a
**4e**	L-Methionine (L-Met)	2.90 ± 0.51	f
**4f**	L-Threonine (L-Thr)	5.80 ± 0.71	def
**4g**	L-Isoleucine (L-Ile)	2.59 ± 0.40	f
**4h**	L-Phenylalanine (L-Phe)	4.76 ± 0.84	ef
**4i**	L-Tryptophan (L-Trp)	ND *^b^*	-
**4j**	L-Tyrosine (L-Tyr)	ND	-
**4k**	L-Glutamic acid (L-Glu)	ND	
**4l**	L-Asparagine (L-Asn)	9.65 ± 0.33	cd
**PCA**	-	ND	-

^a^ Phloem sap collection started at two hours to the end of four hours. Each data point is the mean of 12 seedlings ± SE (n = 3). ^b^ ND means not detected. ^c^ Significant difference means differences between three repetitions of the detected concentration in phloem sap of each conjugate at 5% level.

**Table 4:** Physicochemical properties of compounds **4a**–**l** and PCA.

Compound	Conjugated Amino Acid	Molecular Weight (g/mol)	p*K*a	Log *K*_ow_
**4a**	Glycine (Gly)	281.3	3.29	1.06
**4b**	L-Alanine (L-Ala)	295.3	3.33	1.48
**4c**	L-Leucine (L-Leu)	352.41	3.35	2.42
**4d**	L-Valine (L-Val)	323.35	3.34	1.95
**4e**	L-Methionine (L-Met)	355.41	3.15	2.19
**4f**	L-Threonine (L-Thr)	325.32	2.92	1.34
**4g**	L-Isoleucine (L-Ile)	337.37	3.36	2.59
**4h**	L-Phenylalanine (L-Phe)	371.39	3.13	2.95
**4i**	L-Tryptophan (L-Trp)	410.42	3.32	3.31
**4j**	L-Tyrosine (L-Tyr)	387.39	3.28	2.97
**4k**	L-Glutamic acid (L-Glu)	353.33	3.15	0.70
**4l**	L-Asparagine (L-Asn)	352.34	3.29	0.46
**PCA**	-	224.21	2.34	1.59

Log *K*_ow_ was calculated by the ALOGPS 2.1 program; p*K*a was calculated by the ACD LogDv 6.00 software.

## Supplementary Materials

Supplementary materials are available online.
