# Supplementary material for: Design, Synthesis, Phloem Mobility, and Bioactivities of a Series of Phenazine-1-Carboxylic Acid-Amino Acid Conjugates
Source: Molecules. 2018 Aug 25;23(9):2139. doi: 10.3390/molecules23092139 (PMC6225111; doi:10.3390/molecules23092139)
Supplement: Supplementary file 1 [file molecules-23-02139-s001.pdf]

Supplementary Information

Design, synthesis, phloem mobility and bioactivities of a series of phenazine-1-carboxylic acid-amino acid conjugates

Linhua Yu[1,a], Di Huang[1,a], Xiang Zhu[1,a,b], Min Zhang[a], Zongli Yao[a], Qinglai Wu[b], Zhihong Xu[a, b]*, Junkai Li[a, b]*

[a] *College of Agriculture, Yangtze University, Jingmi Road 88, Jingzhou 434025, China*
[b] *Institute of Pesticides, Yangtze University, Jingmi Road 88, Jingzhou 434025, China*

[1] These authors contributed equally to this work.

*Corresponding author. Tel/Fax: +86 716-8066314.

E-mail address: x_u_78@sina.com (Zhihong Xu).

*Corresponding author. Tel/Fax: +86 716-8066767.

E-mail address: junkaili@sina.com (Junkai Li).

Table of Contents

Trace chromatograms of phloem sap sample **4a** ······ **Figure S1**

Trace chromatograms of phloem sap sample **4b** ······ **Figure S2**

Trace chromatograms of phloem sap sample **4c** ······ **Figure S3**

Trace chromatograms of phloem sap sample **4d** ······ **Figure S4**

Trace chromatograms of phloem sap sample **4e** ······ **Figure S5**

Trace chromatograms of phloem sap sample **4f** ······ **Figure S6**

Trace chromatograms of phloem sap sample **4g** ······ **Figure S7**

Trace chromatograms of phloem sap sample **4h** ······ **Figure S8**

Trace chromatograms of phloem sap sample **4l** ······ **Figure S9**

^{1}H-NMR Spectrum of compound **4a** ······ **Figure S10**

^{13}C-NMR Spectrum of compound **4a** ······ **Figure S11**

HRMS Spectrum of compound **4a** ······ **Figure S12**

^{1}H-NMR Spectrum of compound **4b** ······ **Figure S13**

^{13}C-NMR Spectrum of compound **4b** ······ **Figure S14**

HRMS Spectrum of compound **4b** ······ **Figure S15**

^{1}H-NMR Spectrum of compound **4c** ······ **Figure S16**

^{13}C-NMR Spectrum of compound **4c** ······ **Figure S17**

HRMS Spectrum of compound **4c** ······ **Figure S18**

^{1}H-NMR Spectrum of compound **4d** ······ **Figure S19**

^{13}C-NMR Spectrum of compound **4d** ······ **Figure S20**

HRMS Spectrum of compound **4d** ······ **Figure S21**

^{1}H-NMR Spectrum of compound **4e** ······ **Figure S22**

^{13}C-NMR Spectrum of compound **4e** ······ **Figure S23**

HRMS Spectrum of compound **4e** ······ **Figure S24**

^{1}H-NMR Spectrum of compound **4f** ······ **Figure S25**

^{13}C-NMR Spectrum of compound **4f** ······ **Figure S26**

HRMS Spectrum of compound **4f** ······ **Figure S27**

^{1}H-NMR Spectrum of compound **4g** ······ **Figure S28**

^{13}C-NMR Spectrum of compound **4g** ··········· **Figure S29**

HRMS Spectrum of compound **4g** ··········· **Figure S30**

^{1}H-NMR Spectrum of compound **4h** ··········· **Figure S31**

^{13}C-NMR Spectrum of compound **4h** ··········· **Figure S32**

HRMS Spectrum of compound **4h** ··········· **Figure S33**

^{1}H-NMR Spectrum of compound **4i** ··········· **Figure S34**

^{13}C-NMR Spectrum of compound **4i** ··········· **Figure S35**

HRMS Spectrum of compound **4i** ··········· **Figure S36**

^{1}H-NMR Spectrum of compound **4j** ··········· **Figure S37**

^{13}C-NMR Spectrum of compound **4i** ··········· **Figure S38**

HRMS Spectrum of compound **4j** ··········· **Figure S39**

^{1}H-NMR Spectrum of compound **4k** ··········· **Figure S40**

^{13}C-NMR Spectrum of compound **4k** ··········· **Figure S41**

HRMS Spectrum of compound **4k** ··········· **Figure S42**

^{1}H-NMR Spectrum of compound **4l** ··········· **Figure S43**

^{13}C-NMR Spectrum of compound **4l** ··········· **Figure S44**

HRMS Spectrum of compound **4l** ··········· **Figure S45**

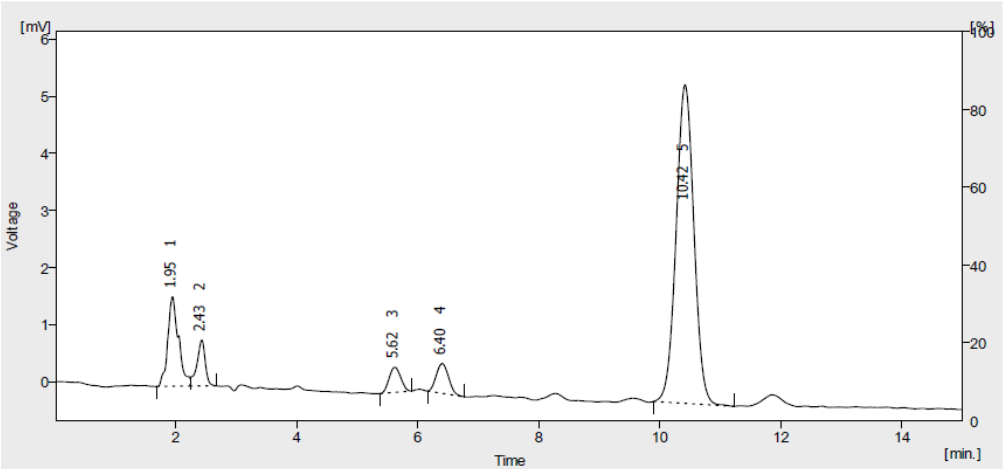

4a-A

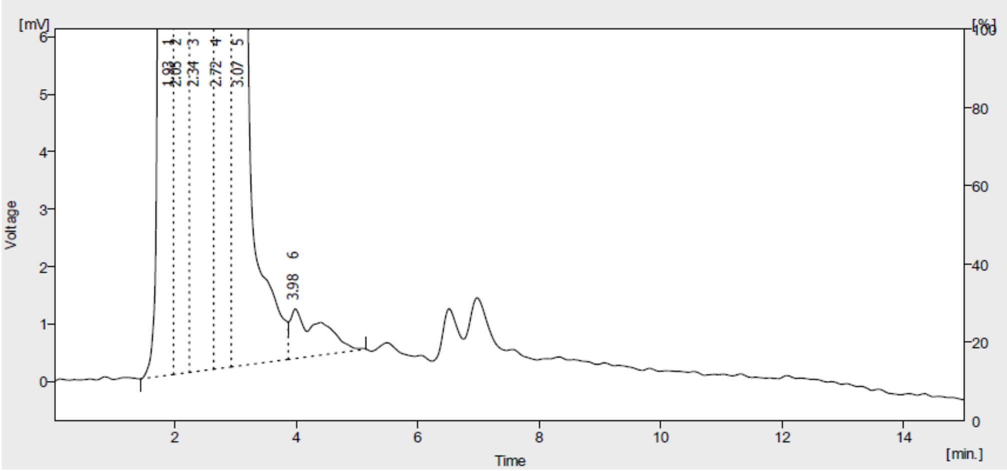

4a-B

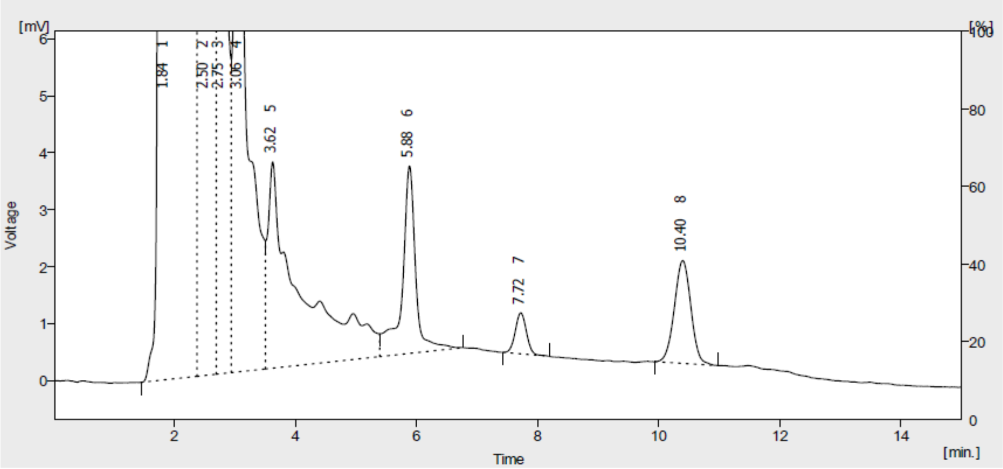

4a-C

Figure S1. *R. communis* sap analysis by HPLC. **4a-A**: standard sample of **4a** (RT: 10.425 min); **4a-B**: control, the cotyledons were incubated in the standard medium; **4a-C**: treated set, the cotyledons were incubated in the same solution with **4a** (RT: 10.408 min) at 0.2 mM concentration.

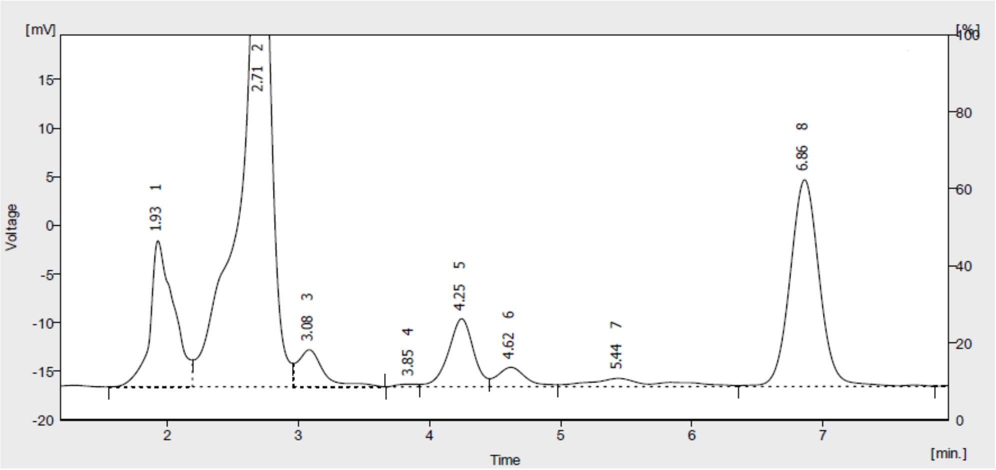

4b-A

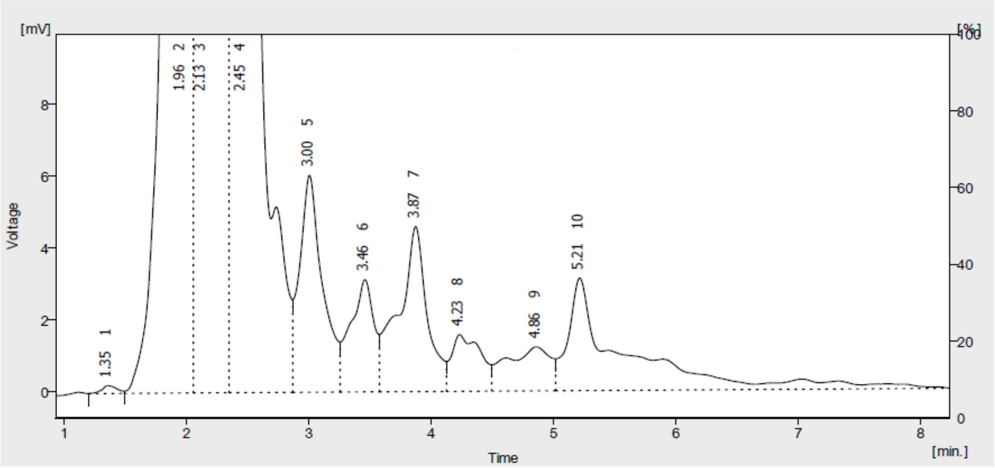

4b-B

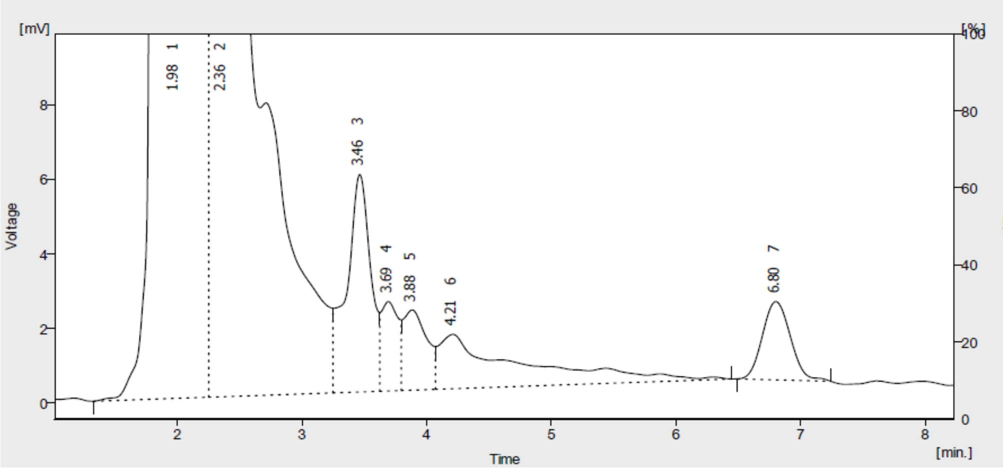

4b-C

Figure S2. *R. communis* sap analysis by HPLC. **4b-A**: standard sample of **4b** (RT: 6.868 min); **4b-B**: control, the cotyledons were incubated in the standard medium; **4b-C**: treated set, the cotyledons were incubated in the same solution with **4b** (RT: 6.807 min) at 0.2 mM concentration.

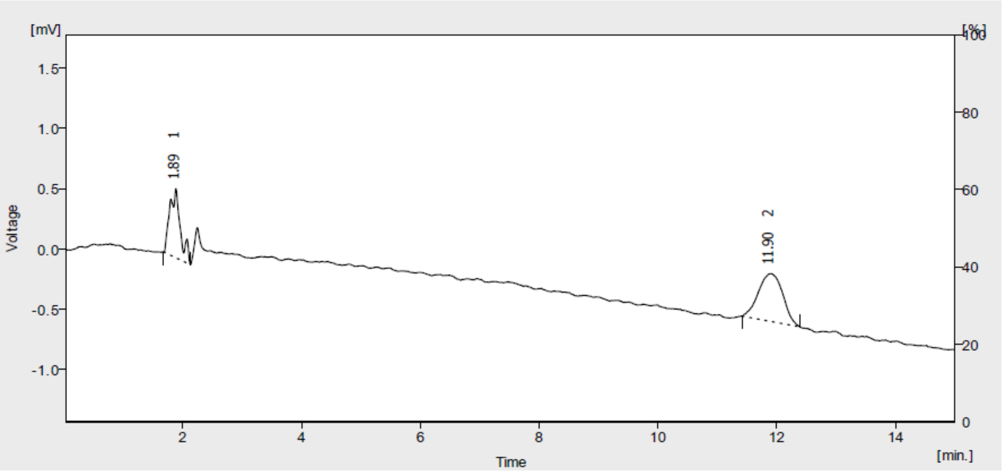

4c-A

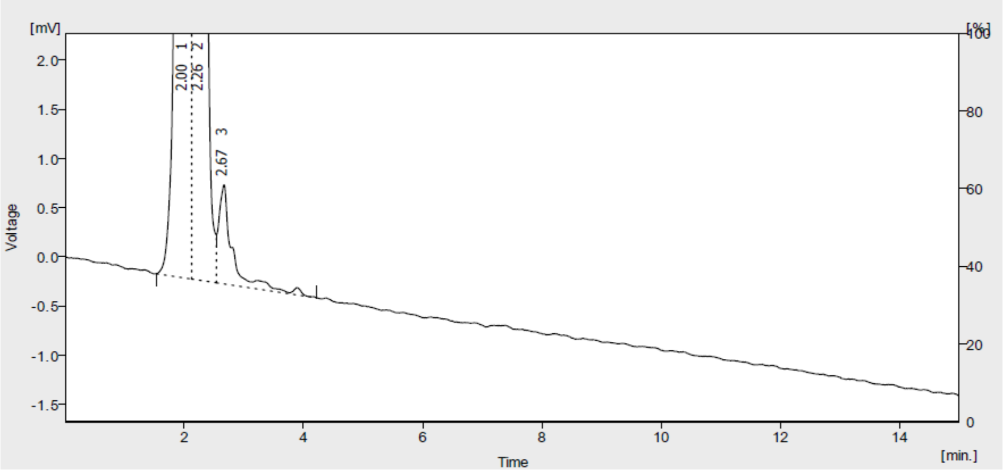

4c-B

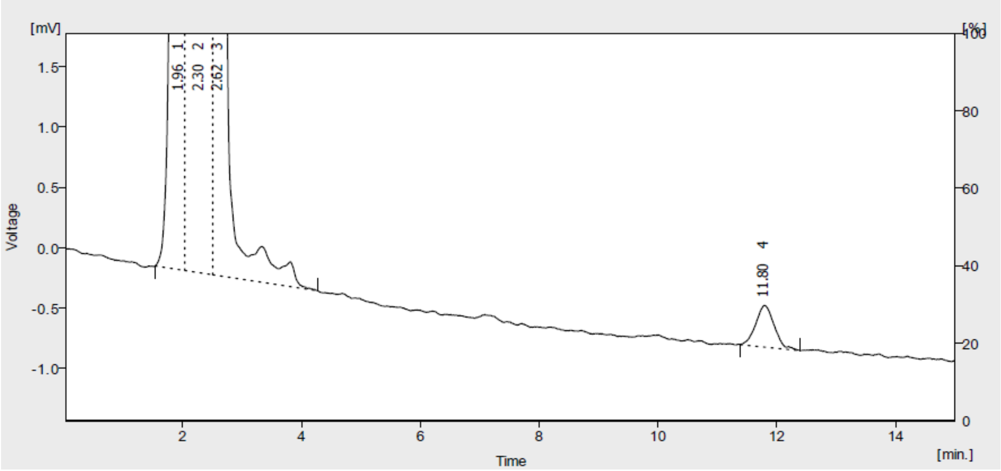

4c-C

Figure S3. *R. communis* sap analysis by HPLC. **4c-A**: standard sample of **4c** (RT: 11.902 min); **4c-B**: control, the cotyledons were incubated in the standard medium; **4c-C**: treated set, the cotyledons were incubated in the same solution with **4c** (RT: 11.804 min) at 0.2 mM concentration.

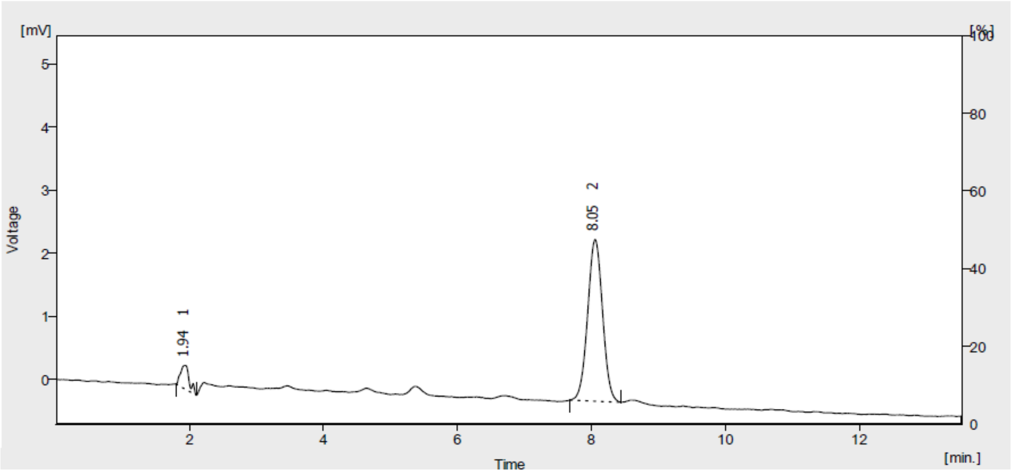

4d-A

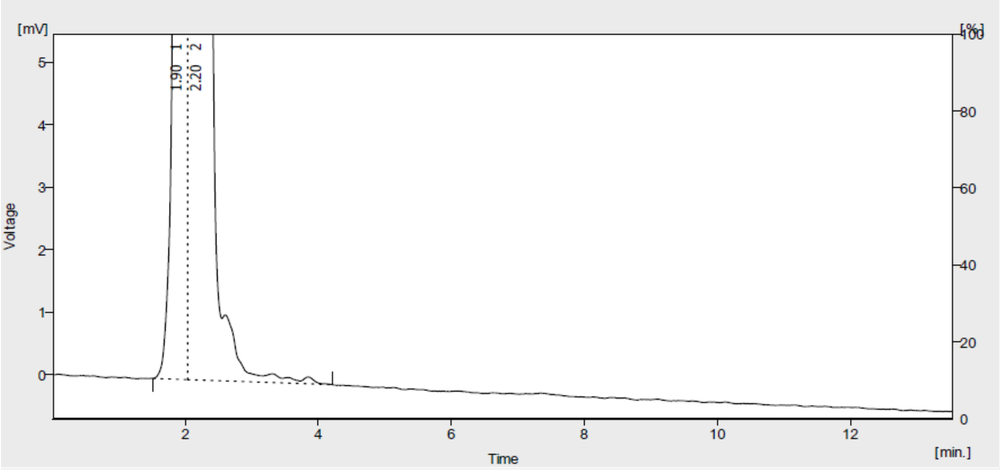

4d-B

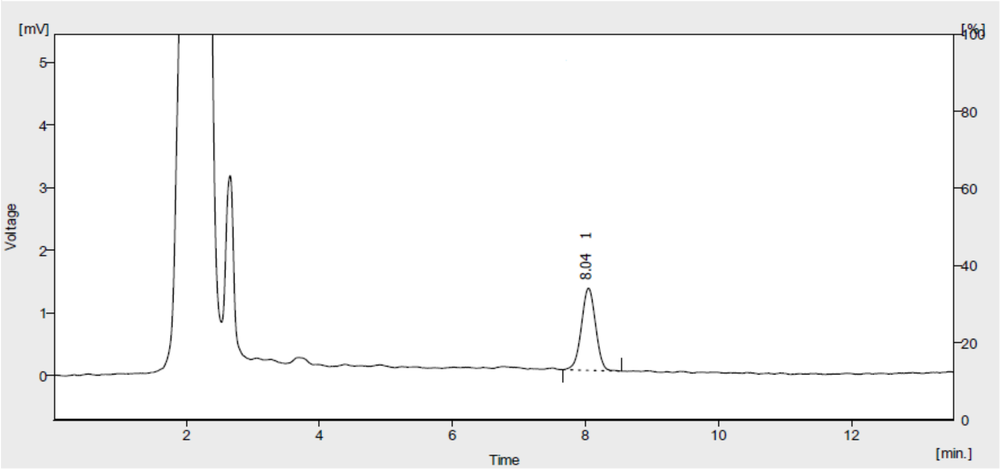

4d-C

Figure S4. *R. communis* sap analysis by HPLC. **4d-A**: standard sample of **4d** (RT: 8.052 min); **4d-B**: control, the cotyledons were incubated in the standard medium; **4d-C**: treated set, the cotyledons were incubated in the same solution with **4d** (RT: 8.041 min) at 0.2 mM concentration.

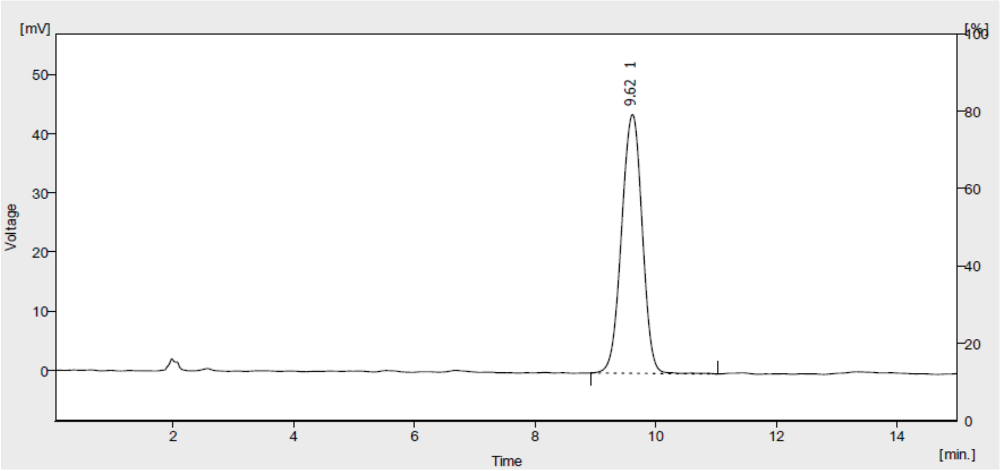

4e-A

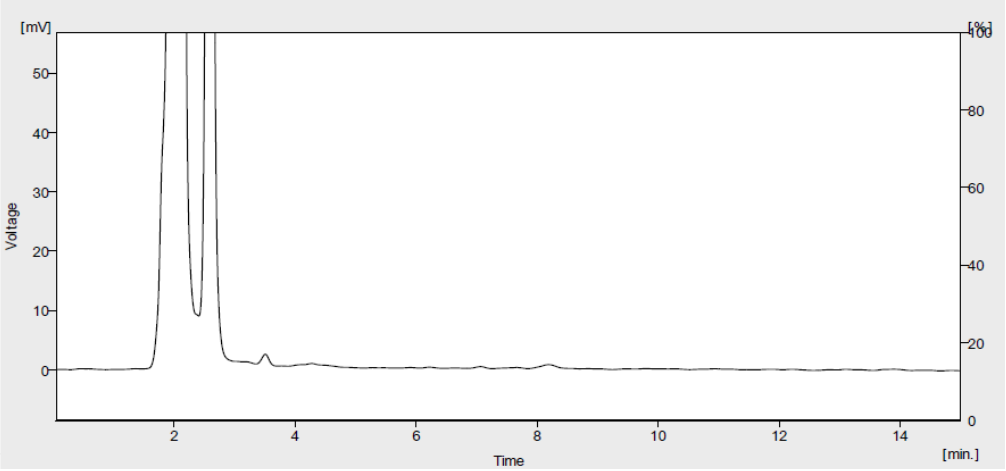

4e-B

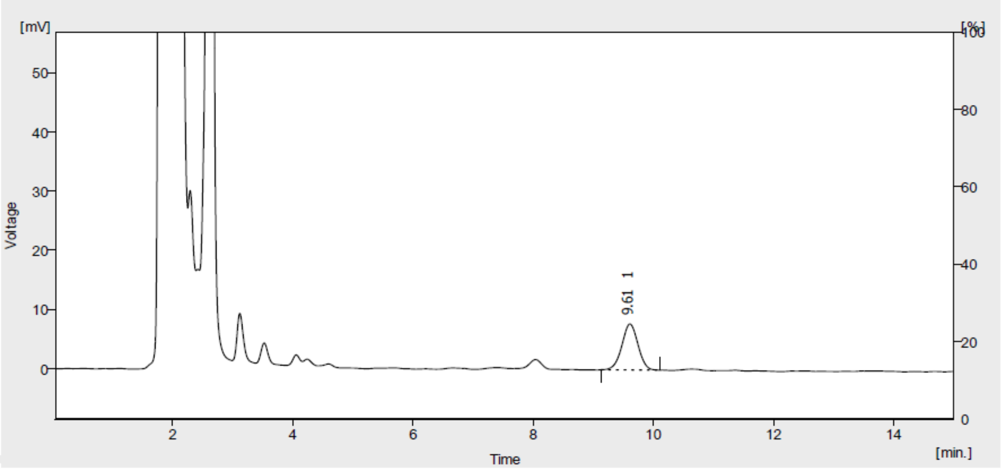

4e-C

Figure S5. *R. communis* sap analysis by HPLC. **4e-A**: standard sample of **4e** (RT: 9.621 min); **4e-B**: control, the cotyledons were incubated in the standard medium; **4e-C**: treated set, the cotyledons were incubated in the same solution with **4e** (RT: 9.611 min) at 0.2 mM concentration.

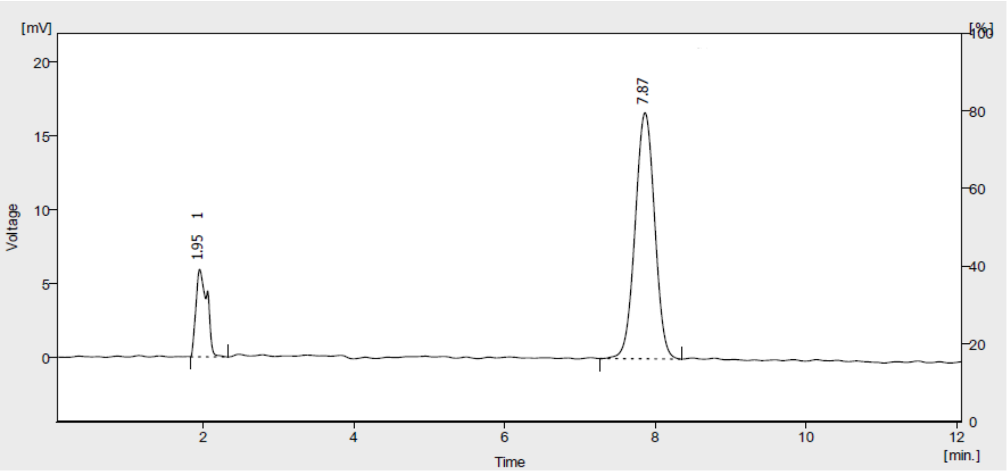

4f-A

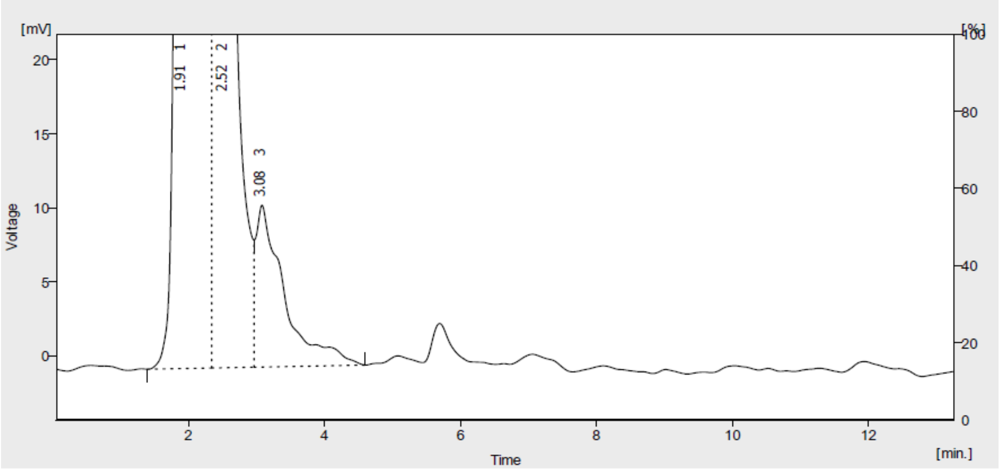

4f-B

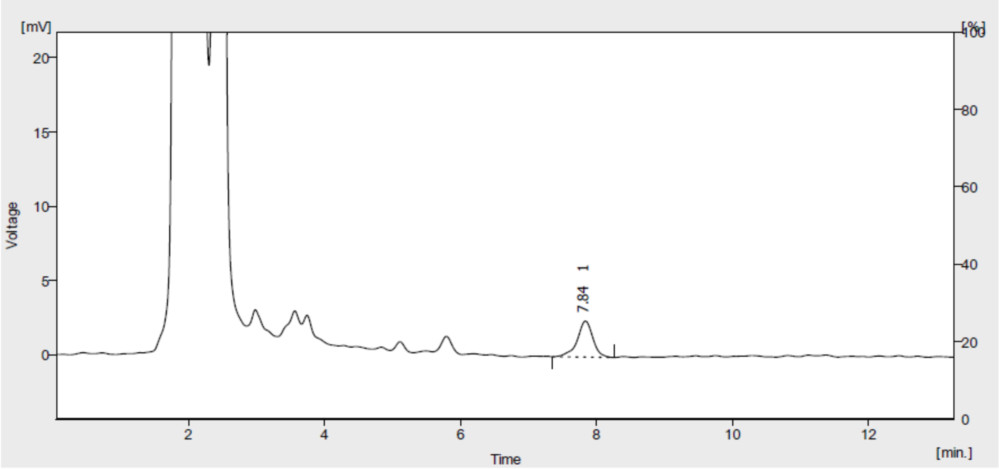

4f-C

Figure S6. *R. communis* sap analysis by HPLC. **4f-A**: standard sample of **4f** (RT: 7.870 min); **4f-B**: control, the cotyledons were incubated in the standard medium; **4f-C**: treated set, the cotyledons were incubated in the same solution with **4f** (RT: 7.841 min) at 0.2 mM concentration.

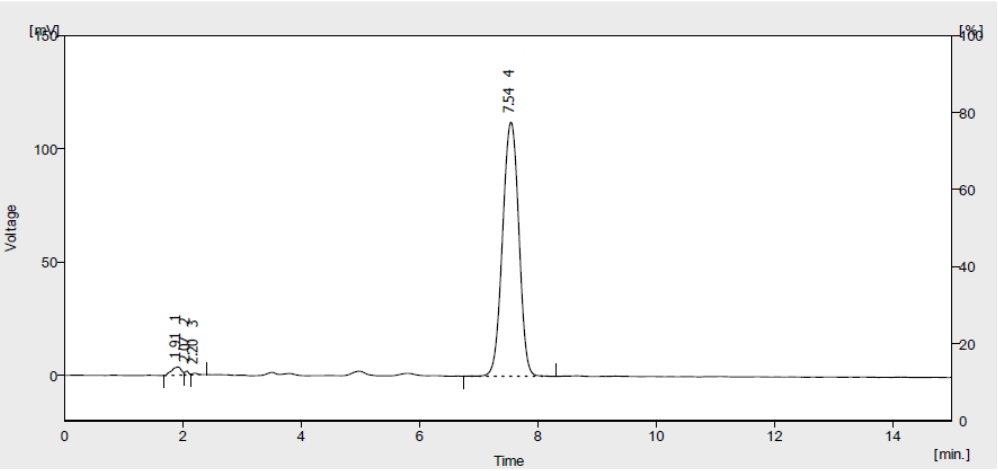

4g-A

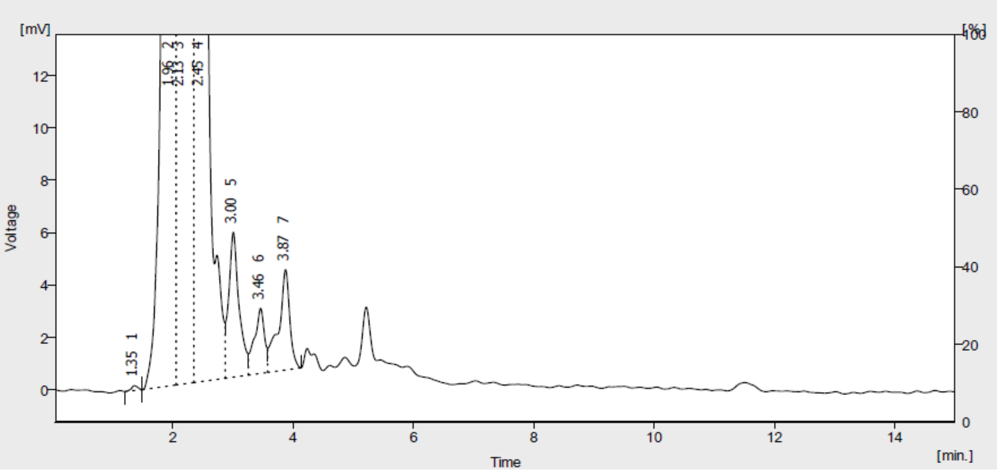

4g-B

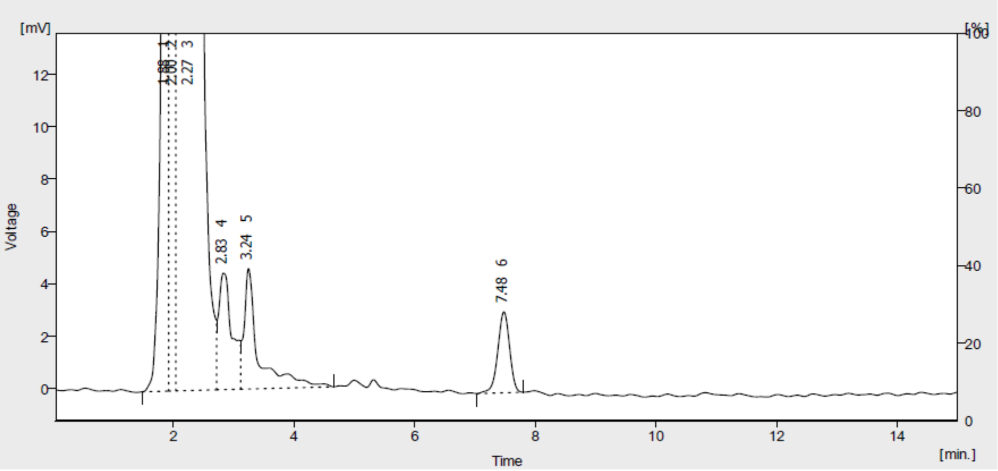

4g-C

Figure S7. *R. communis* sap analysis by HPLC. **4g-A**: standard sample of **4g** (RT: 7.544 min); **4g-B**: control, the cotyledons were incubated in the standard medium; **4g-C**: treated set, the cotyledons were incubated in the same solution with **4g** (RT: 7.486 min) at 0.2 mM concentration.

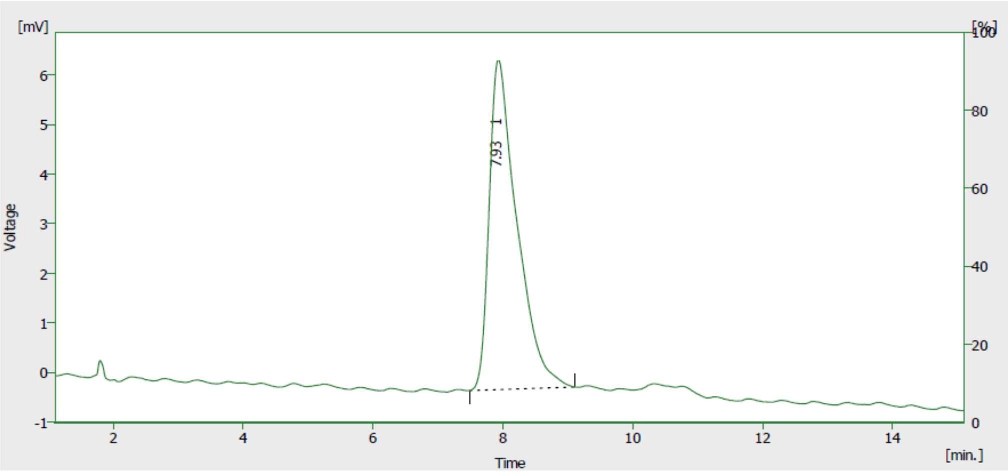

4h-A

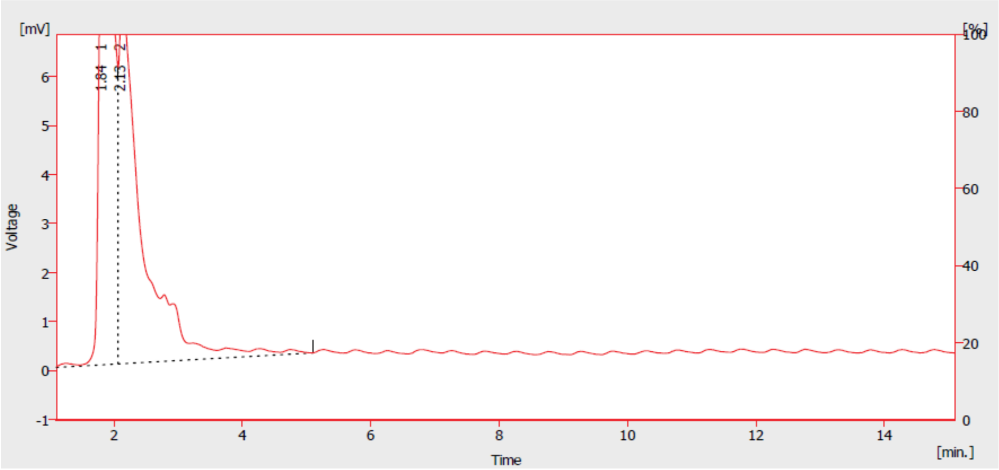

4h-B

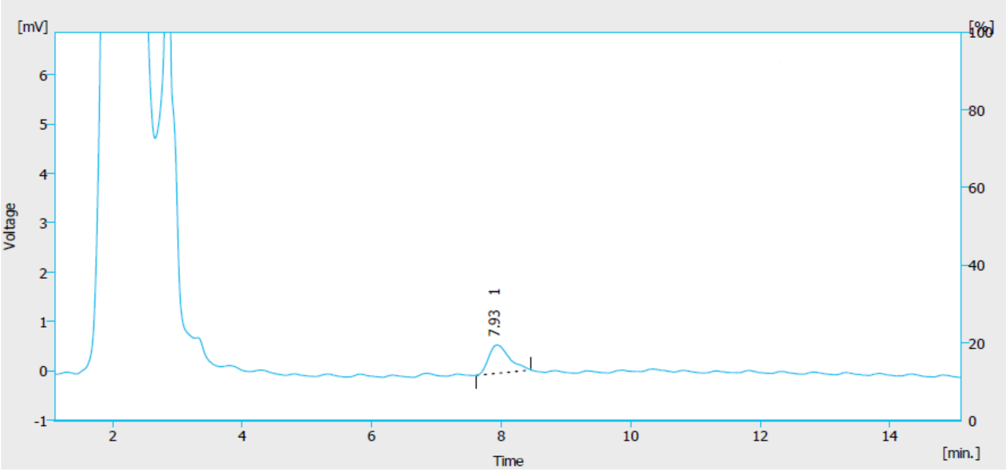

4h-C

Figure S8. *R. communis* sap analysis by HPLC. **4h-A**: standard sample of **4h** (RT: 7.931 min); **4h-B**: control, the cotyledons were incubated in the standard medium; **4h-C**: treated set, the cotyledons were incubated in the same solution with **4h** (RT: 7.931 min) at 0.2 mM concentration.

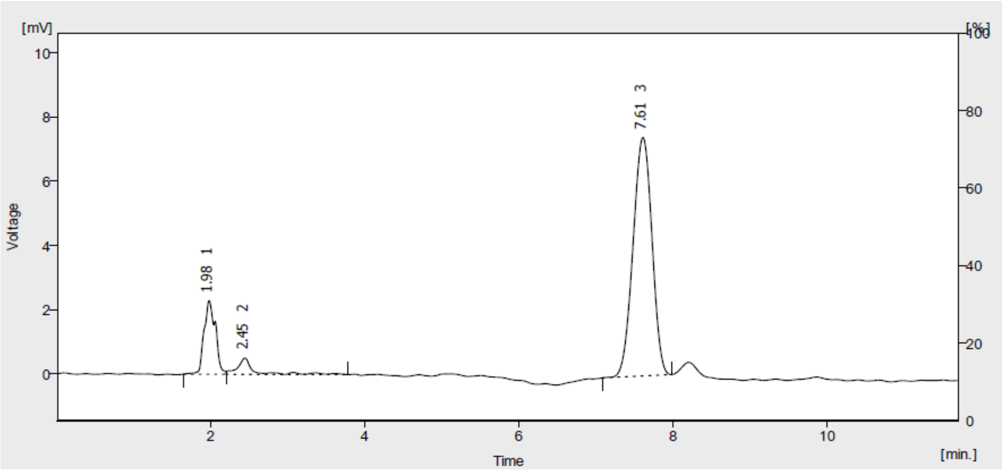

4l-A

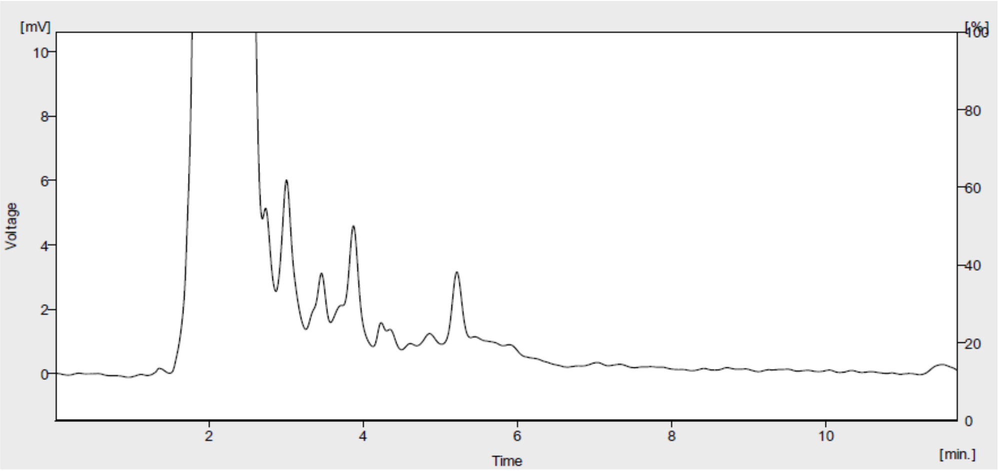

4l-B

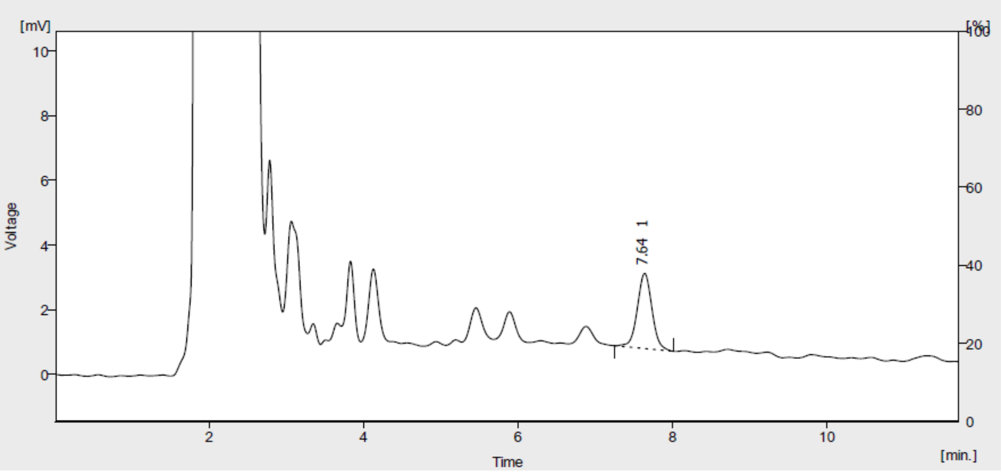

4l-C

Figure S9. *R. communis* sap analysis by HPLC. **4l-A**: standard sample of **4l** (RT: 7.613 min); **4l-B**: control, the cotyledons were incubated in the standard medium; **4l-C**: treated set, the cotyledons were incubated in the same solution with **4l** (RT: 7.641 min) at 0.2 mM concentration.

The compounds **4i**, **4j** and **4k** are not detected in phloem sap of *R. communis.*

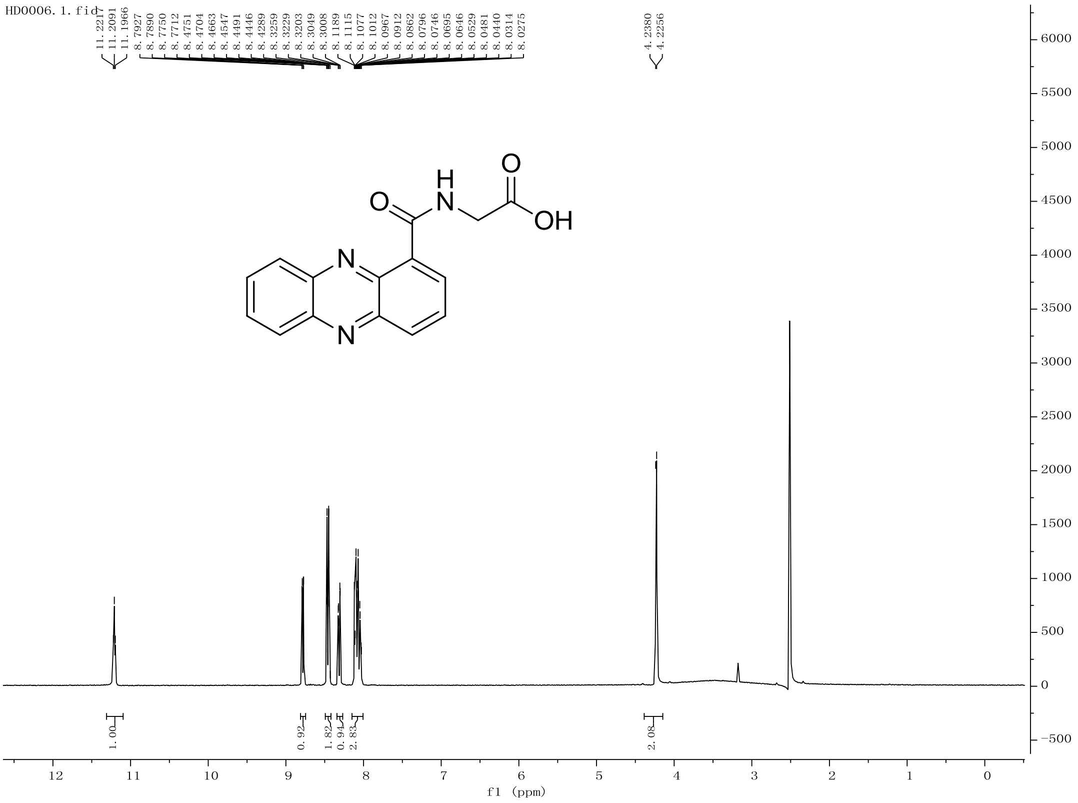

Figure S10. ^{1}H-NMR Spectrum of compound **4a**

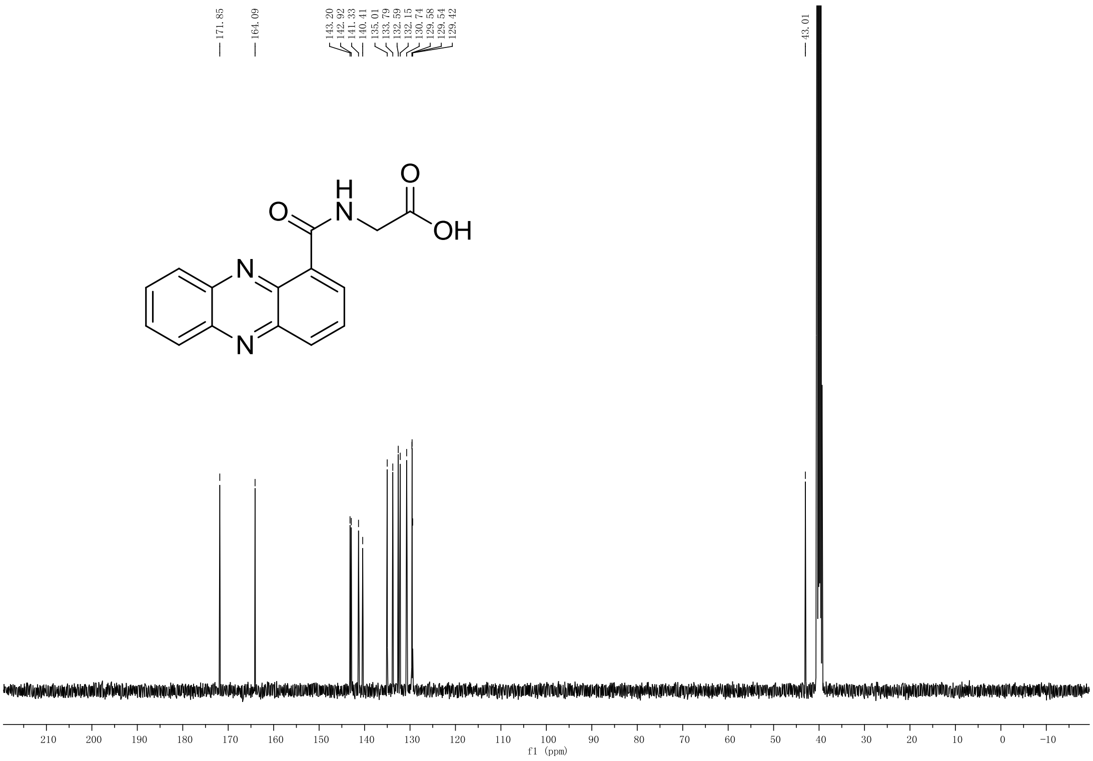

Figure S11. ^{13}C-NMR Spectrum of compound **4a**

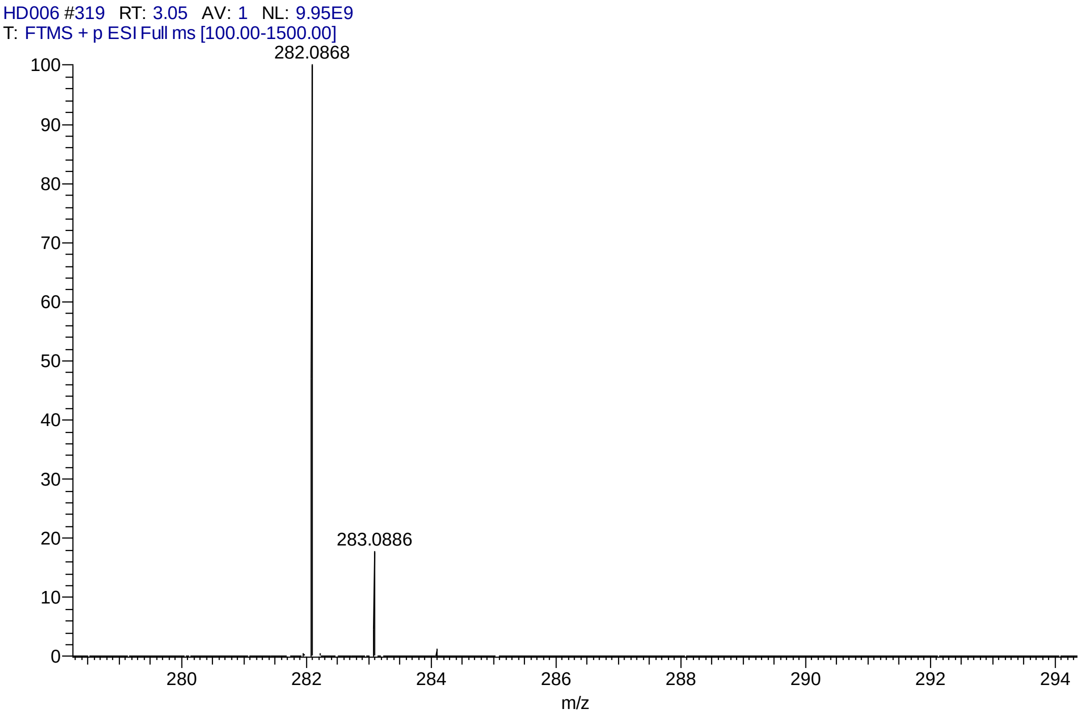

Figure S12. HRMS Spectrum of compound **4a**

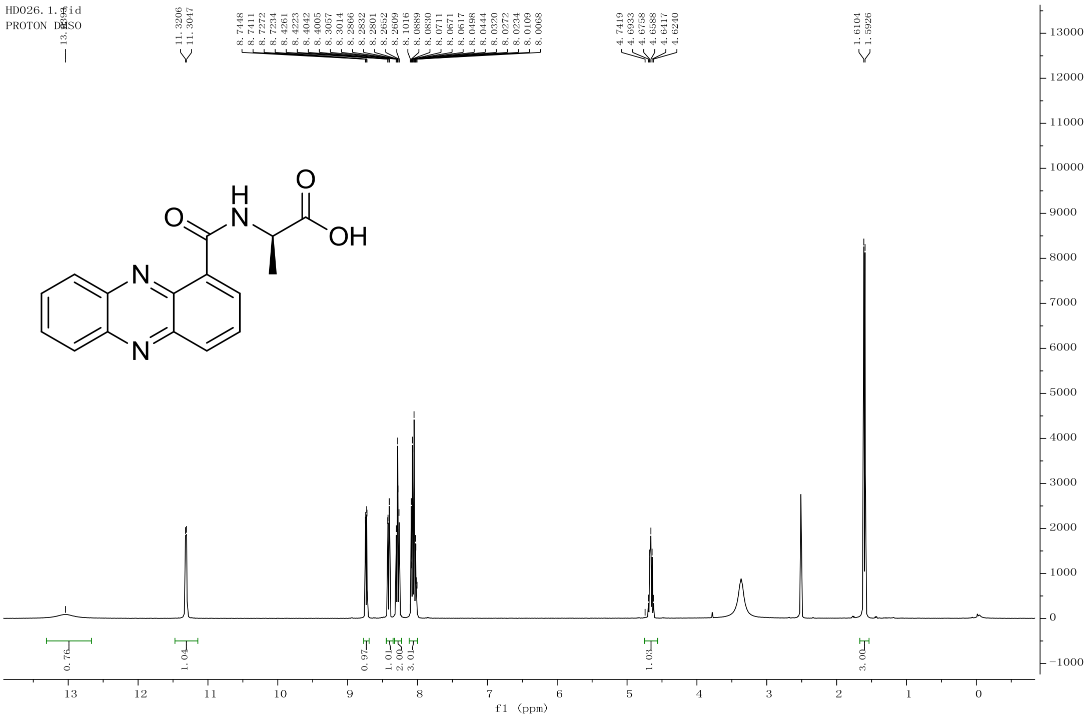

Figure S13. ^{1}H-NMR Spectrum of compound **4b**

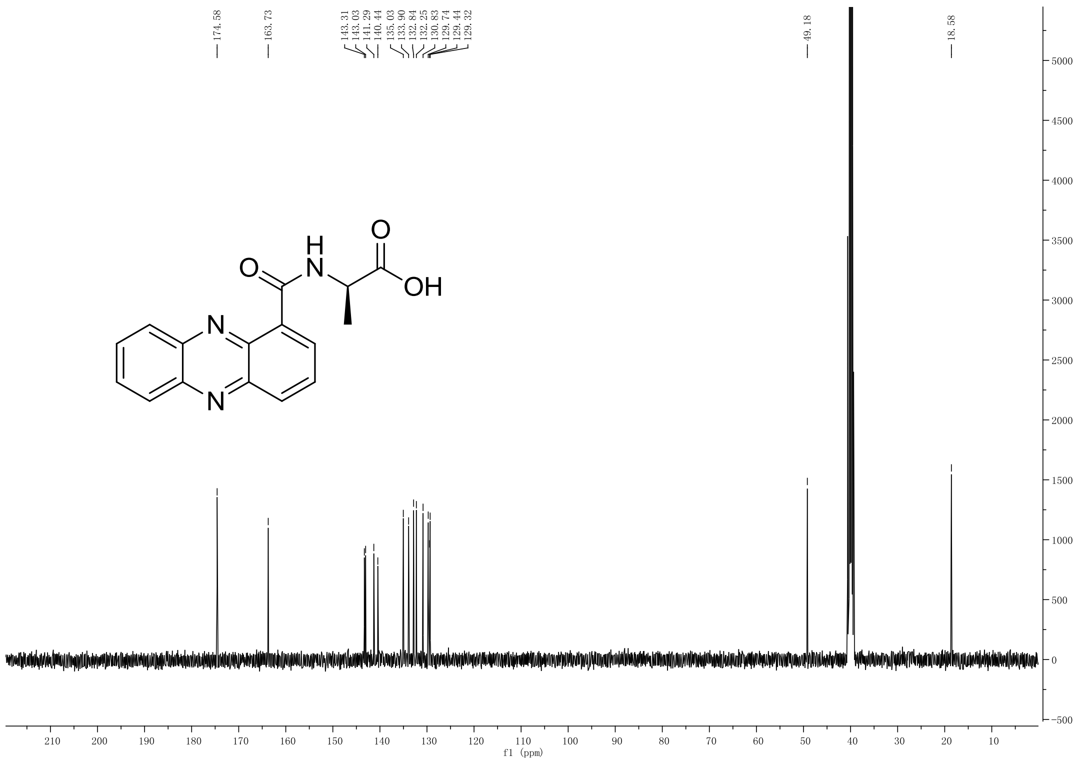

Figure S14. ^{13}C-NMR Spectrum of compound **4b**

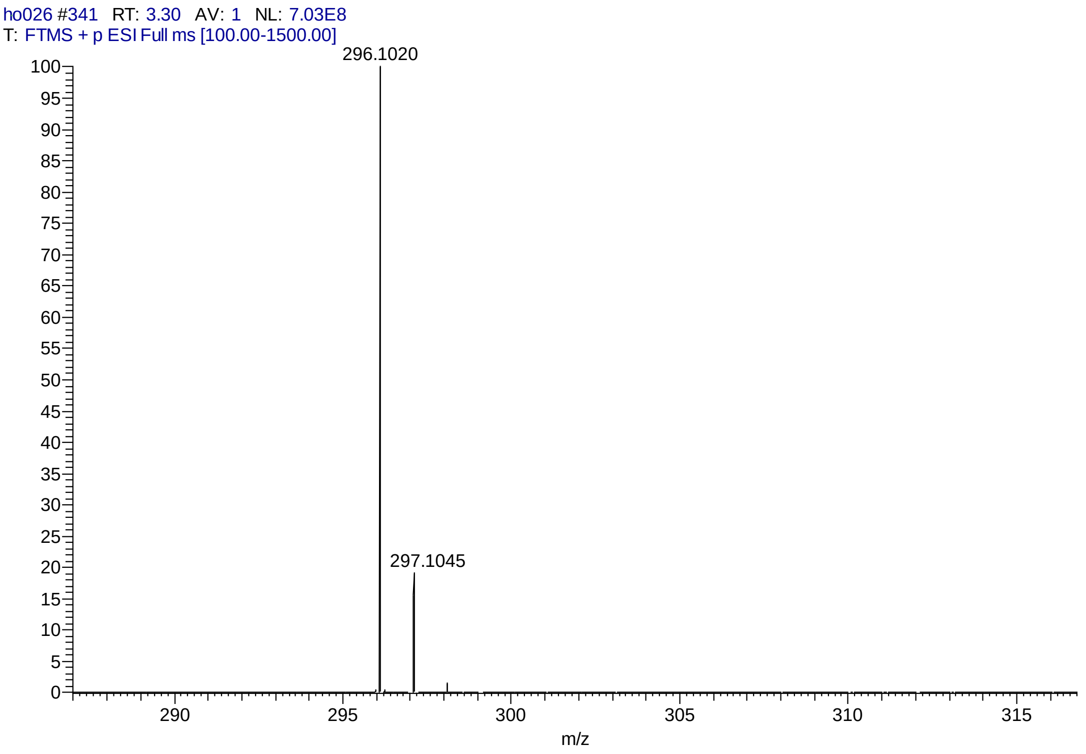

Figure S15. HRMS Spectrum of compound **4b**

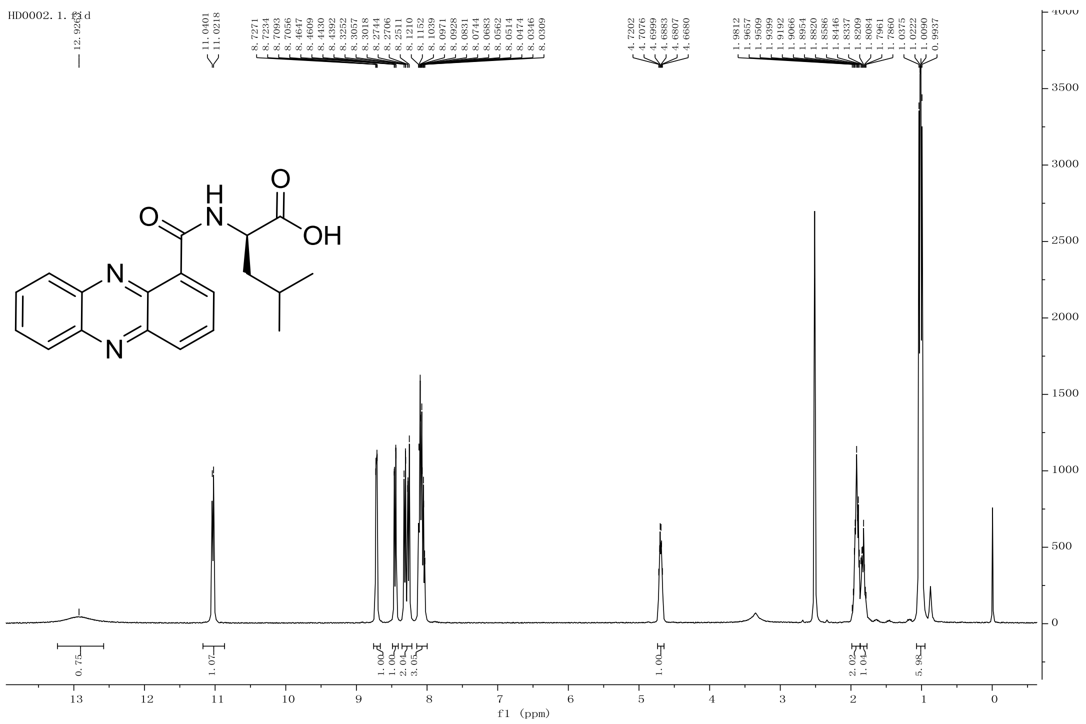

Figure S16. ^{1}H-NMR Spectrum of compound **4c**

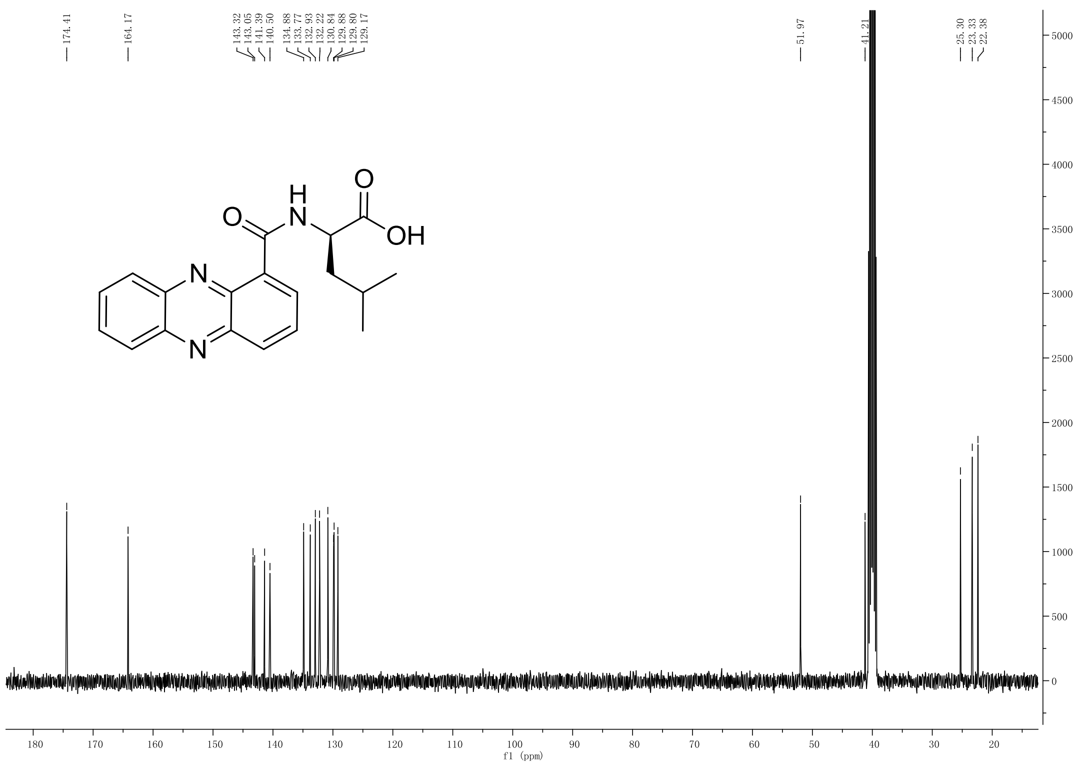

Figure S17. ^{13}C-NMR Spectrum of compound **4c**

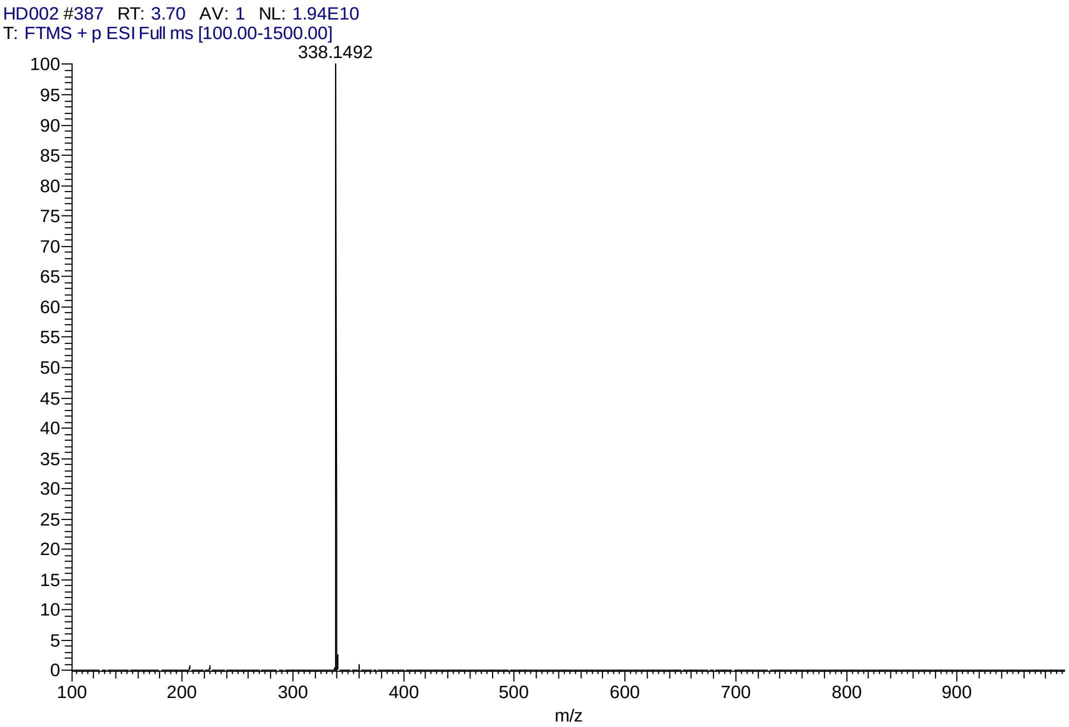

Figure S18. HRMS Spectrum of compound **4c**

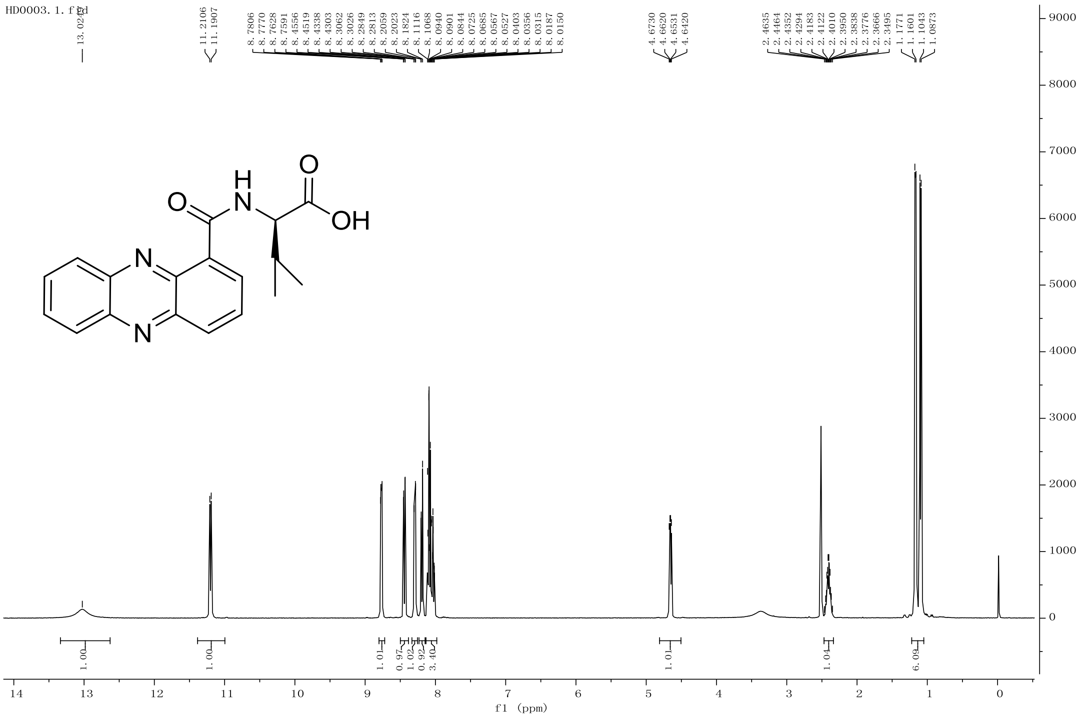

Figure S19. ^{1}H-NMR Spectrum of compound **4d**

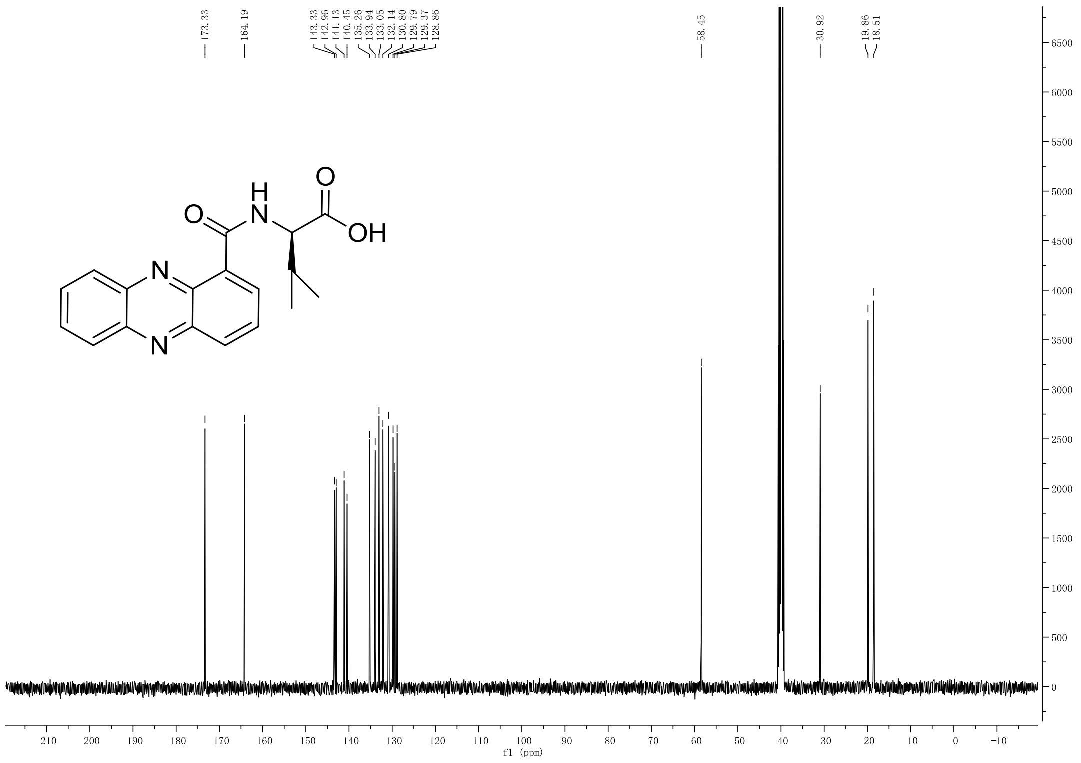

Figure S20. ^{13}C-NMR Spectrum of compound **4d**

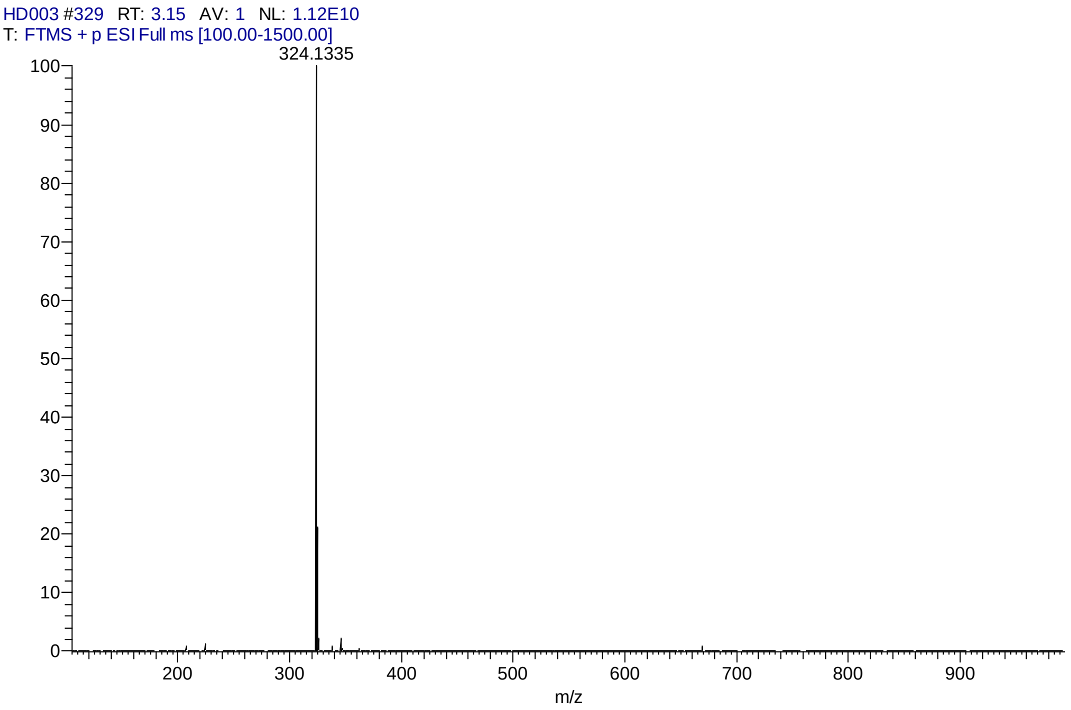

Figure S21. HRMS Spectrum of compound **4d**

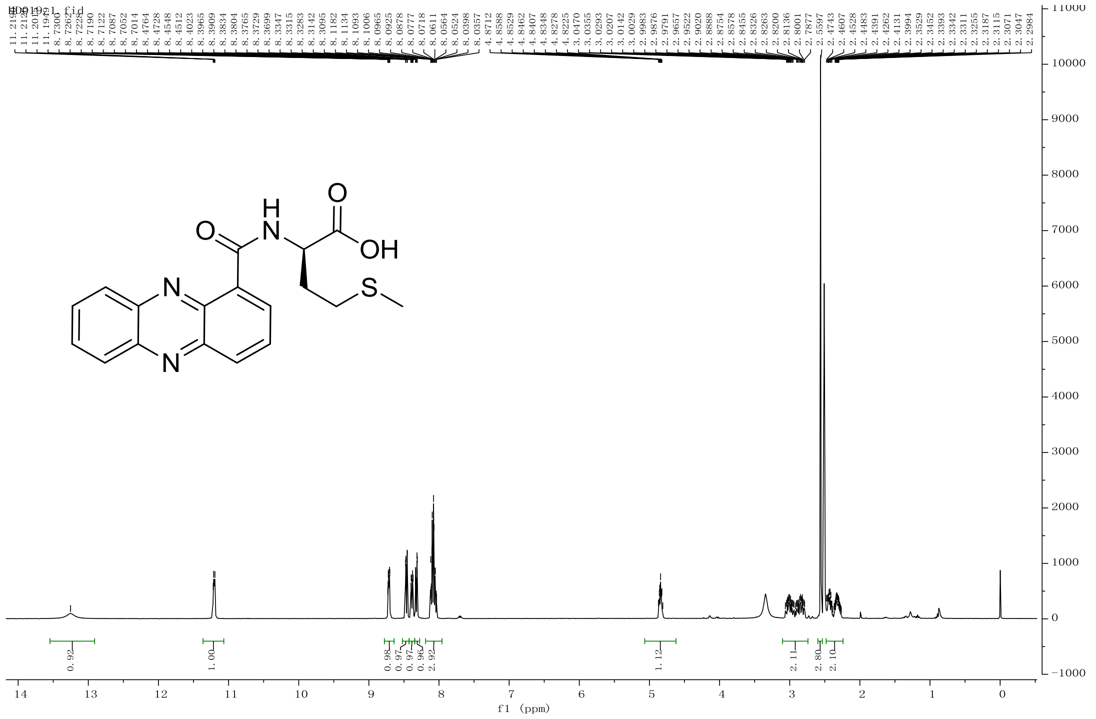

Figure S22. ^{1}H-NMR Spectrum of compound **4e**

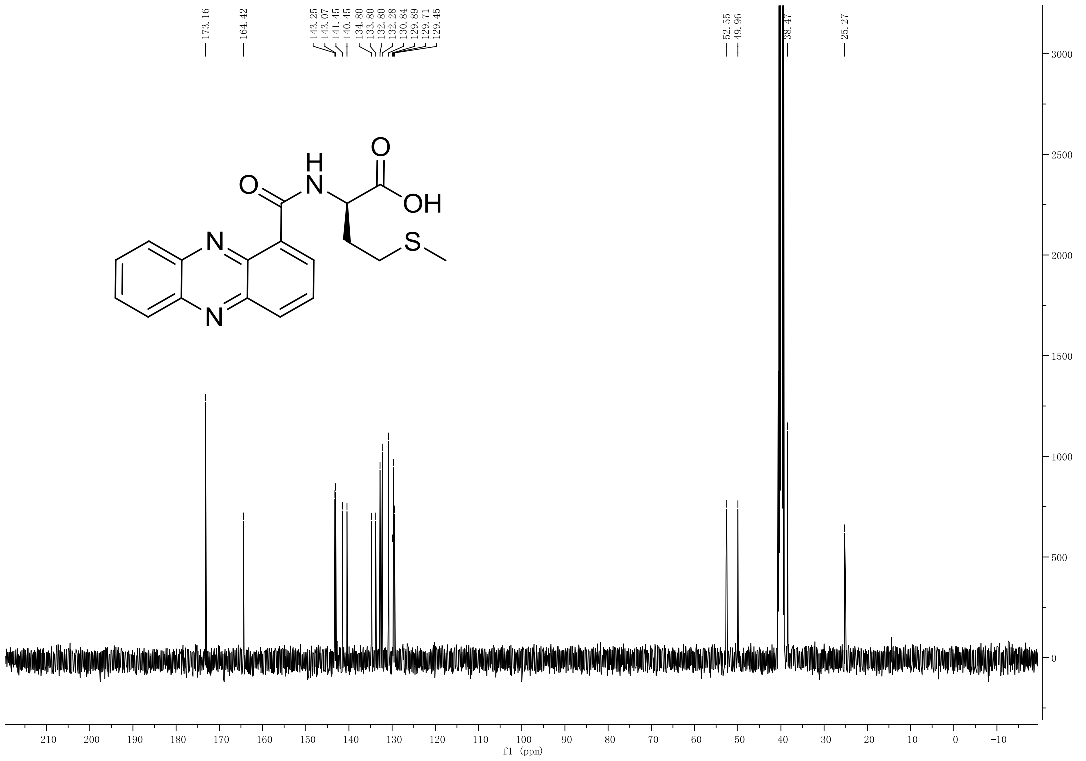

Figure S23. ^{13}C-NMR Spectrum of compound **4e**

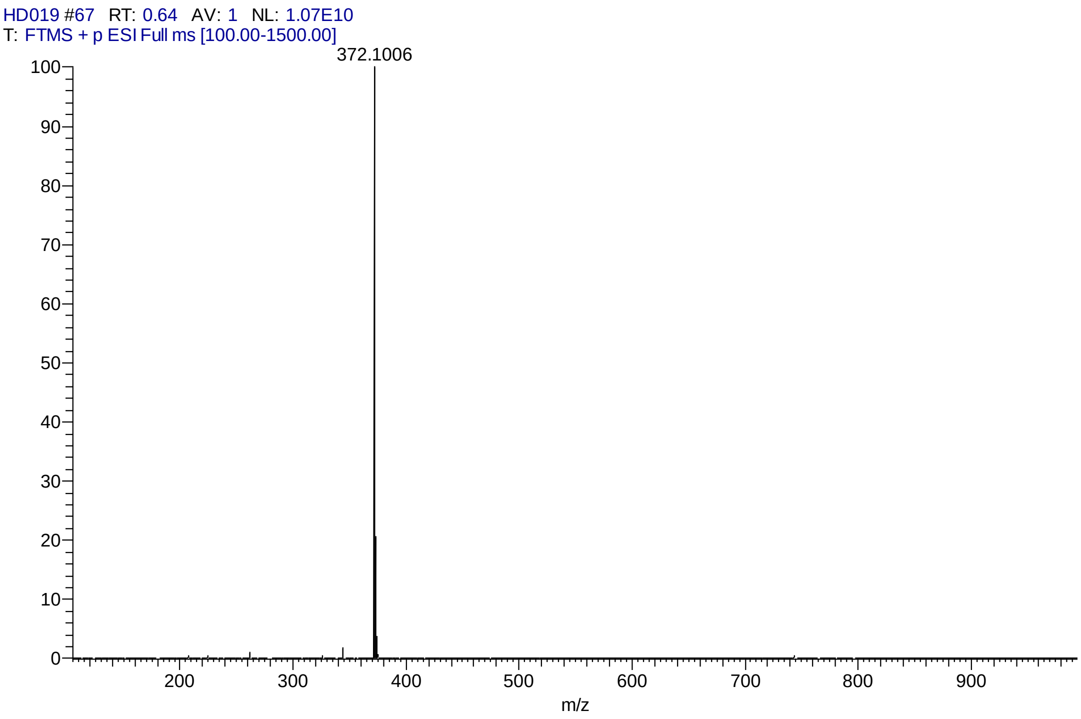

Figure S24. HRMS Spectrum of compound **4e**

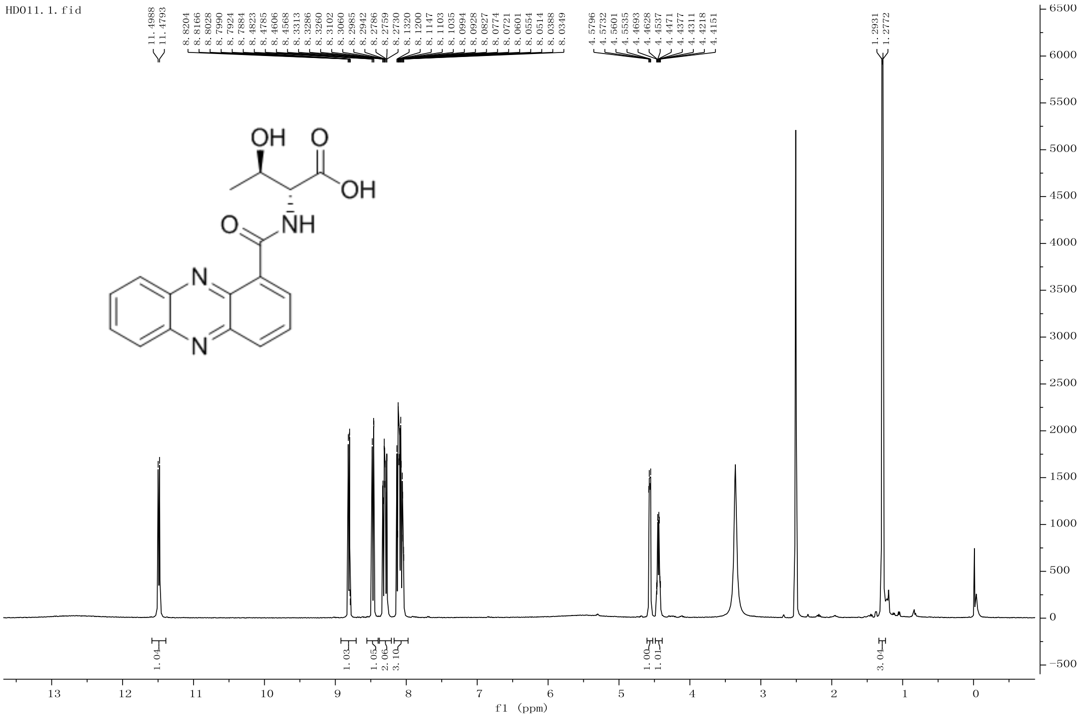

Figure S25. ^{1}H-NMR Spectrum of compound **4f**

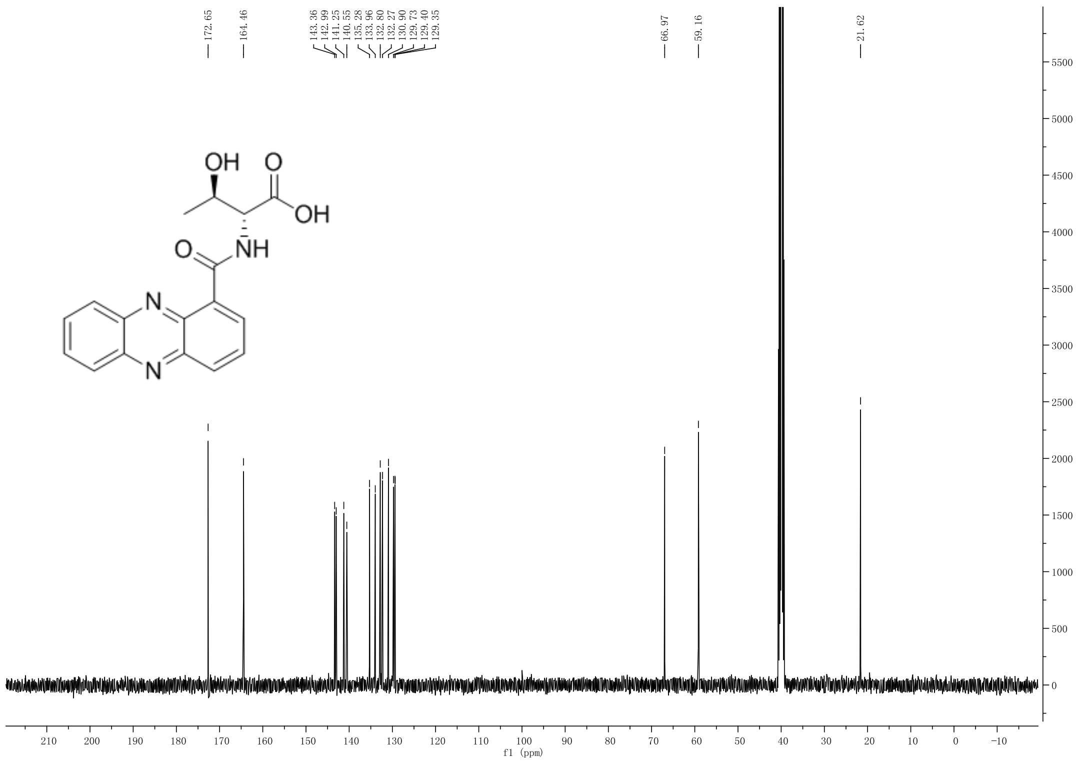

Figure S26. ^{13}C-NMR Spectrum of compound **4f**

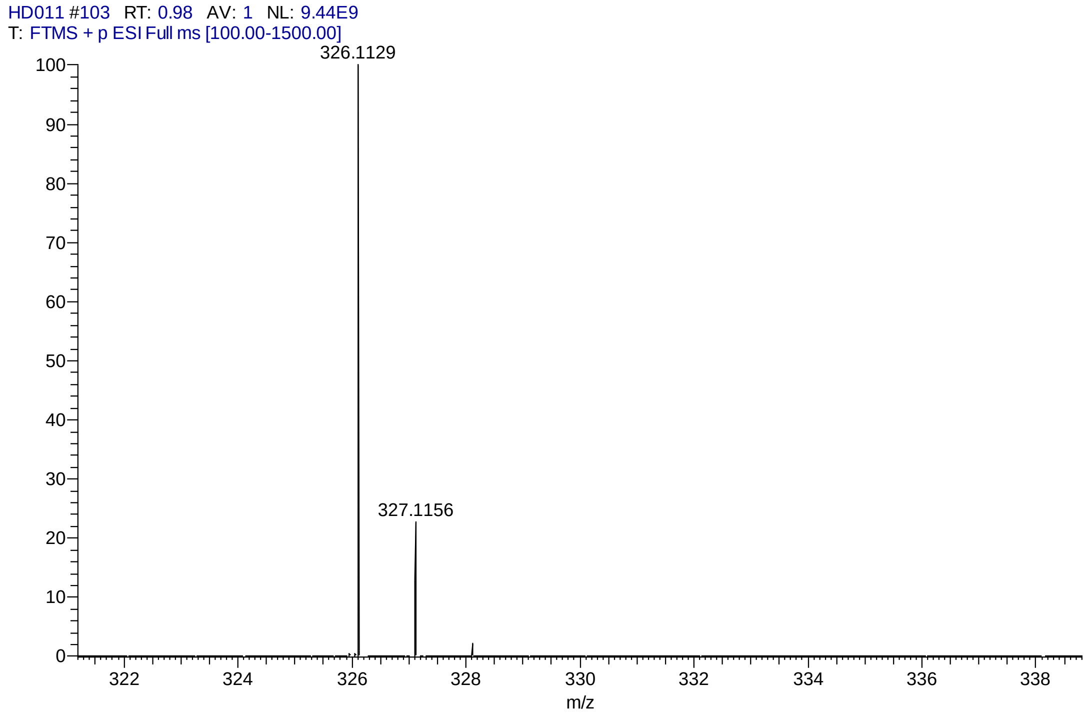

Figure S27. HRMS Spectrum of compound **4f**

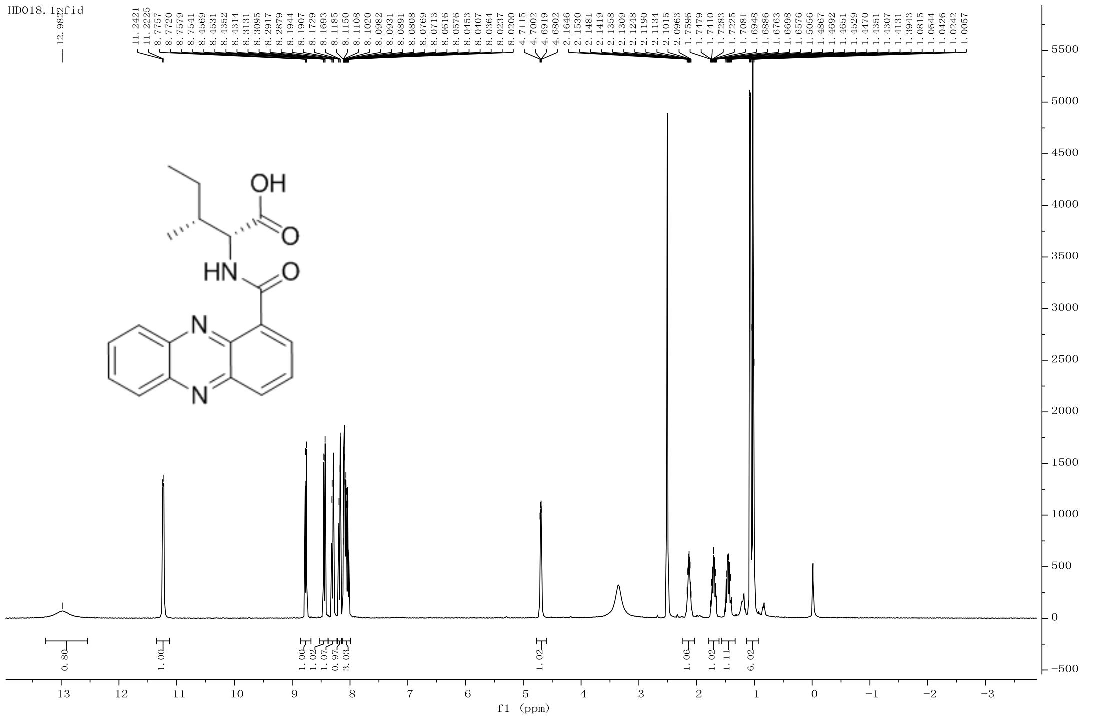

Figure S28. ^{1}H-NMR Spectrum of compound **4g**

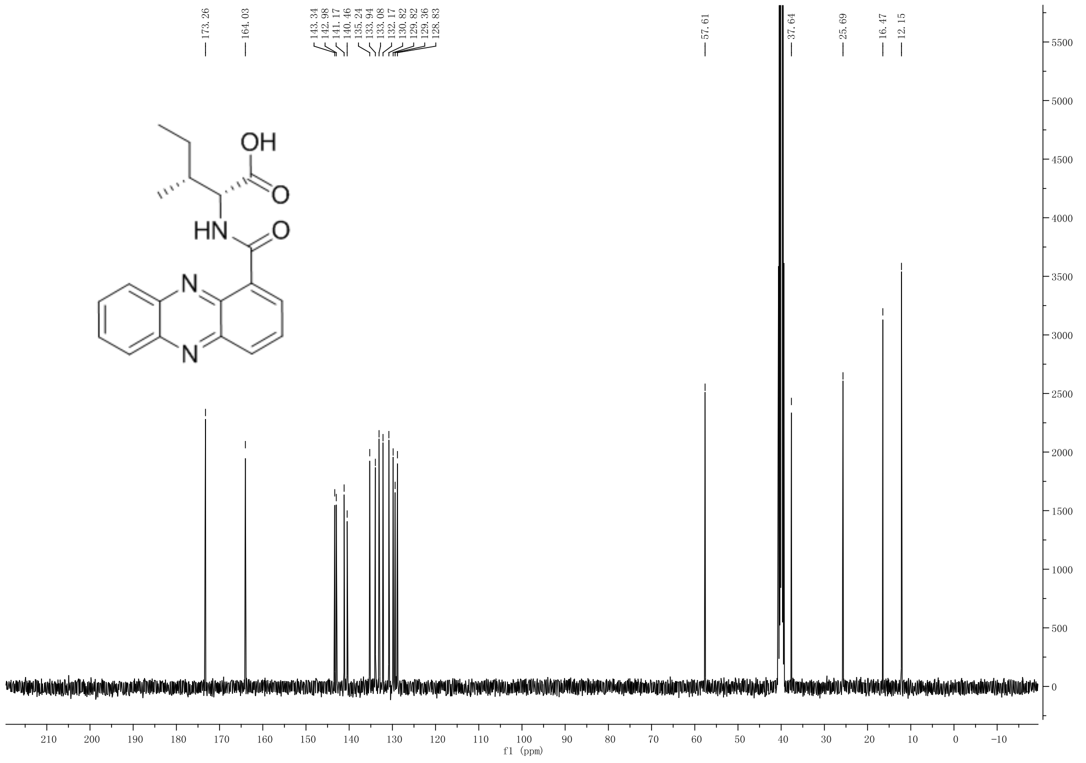

Figure S29. ^{13}C-NMR Spectrum of compound **4g**

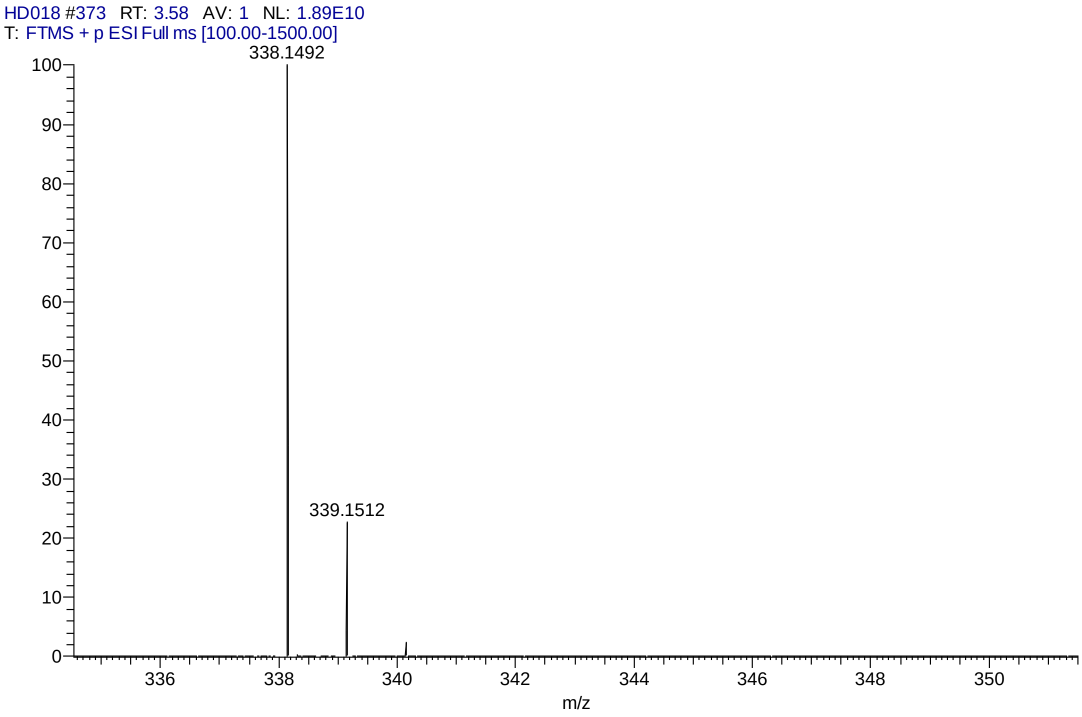

Figure S30. HRMS Spectrum of compound **4g**

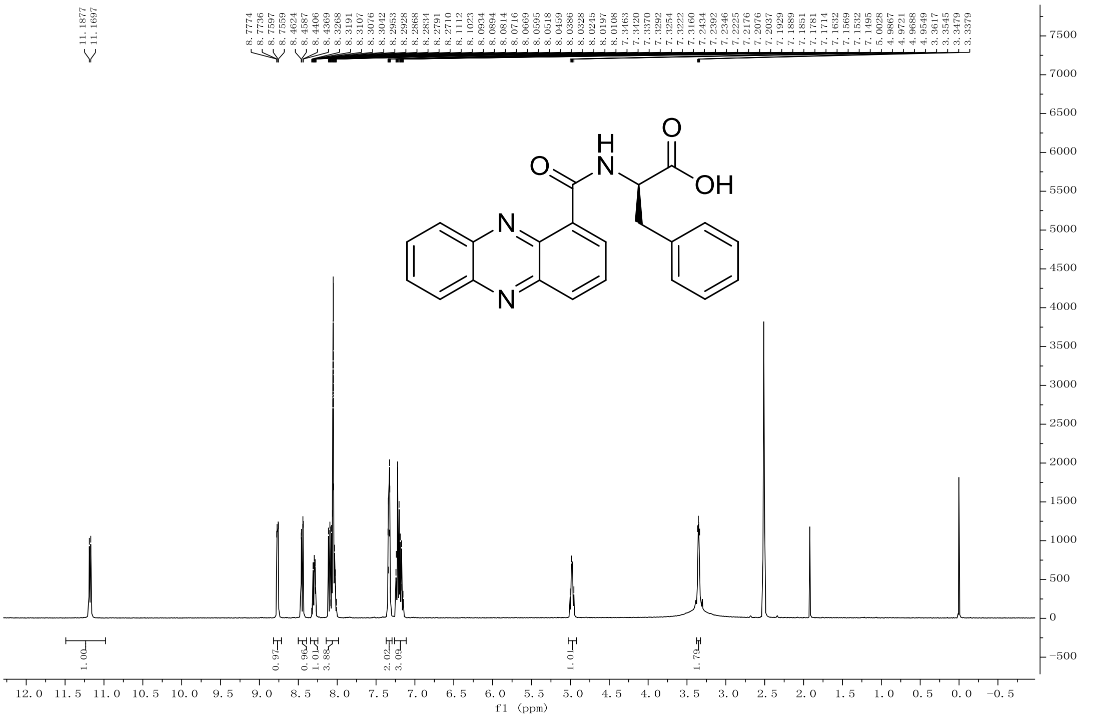

Figure S31. ¹H-NMR Spectrum of compound **4h**

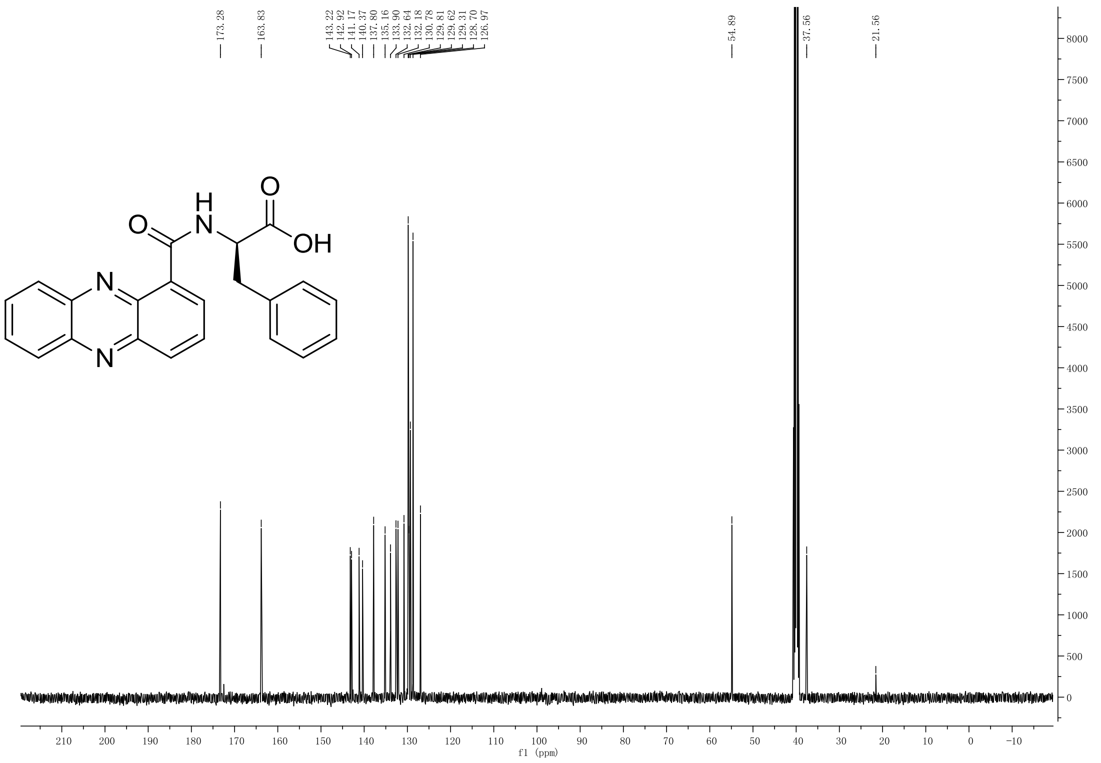

Figure S32. ^{13}C-NMR Spectrum of compound **4h**

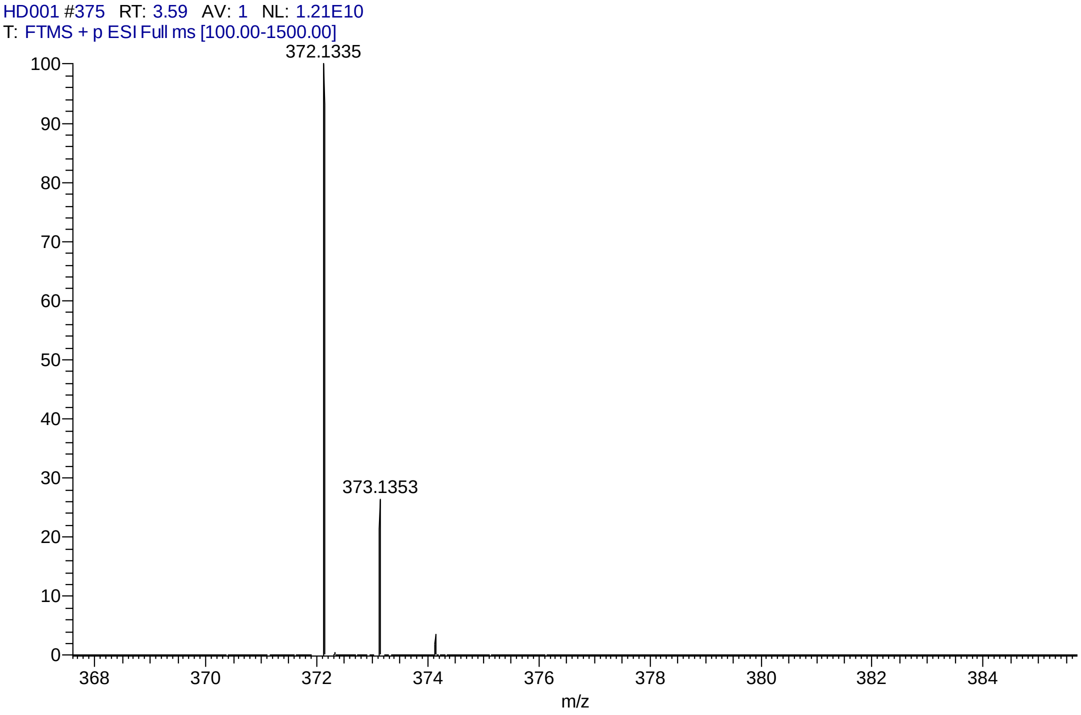

Figure S33. HRMS Spectrum of compound **4h**

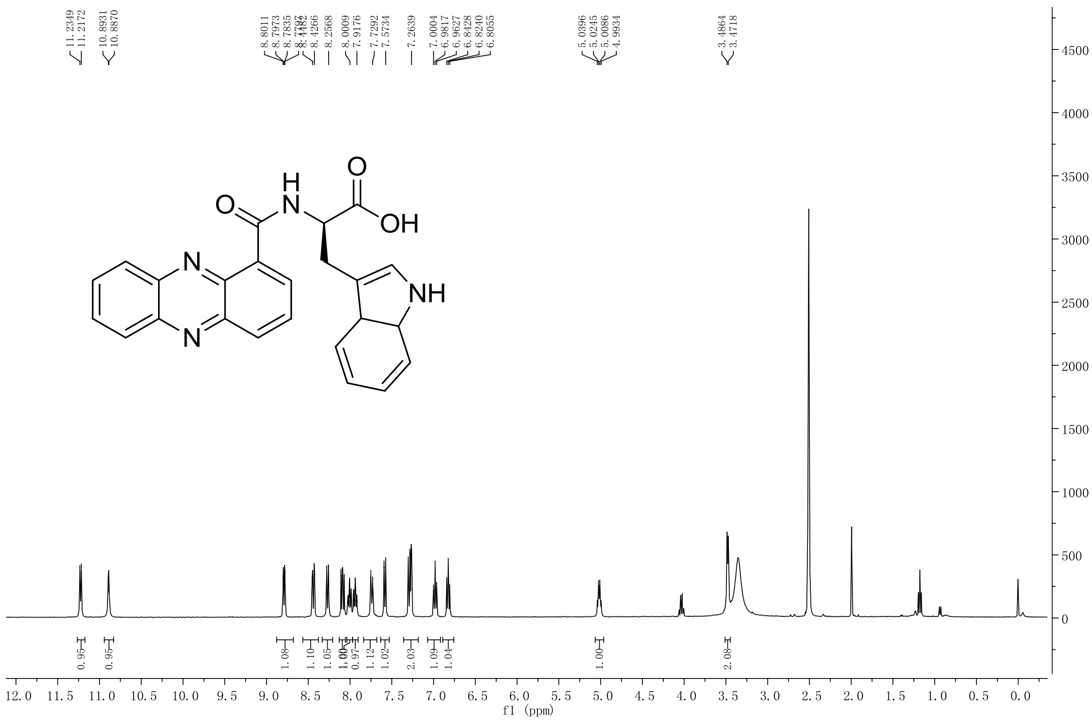

Figure S34. ^{1}H-NMR Spectrum of compound **4i**

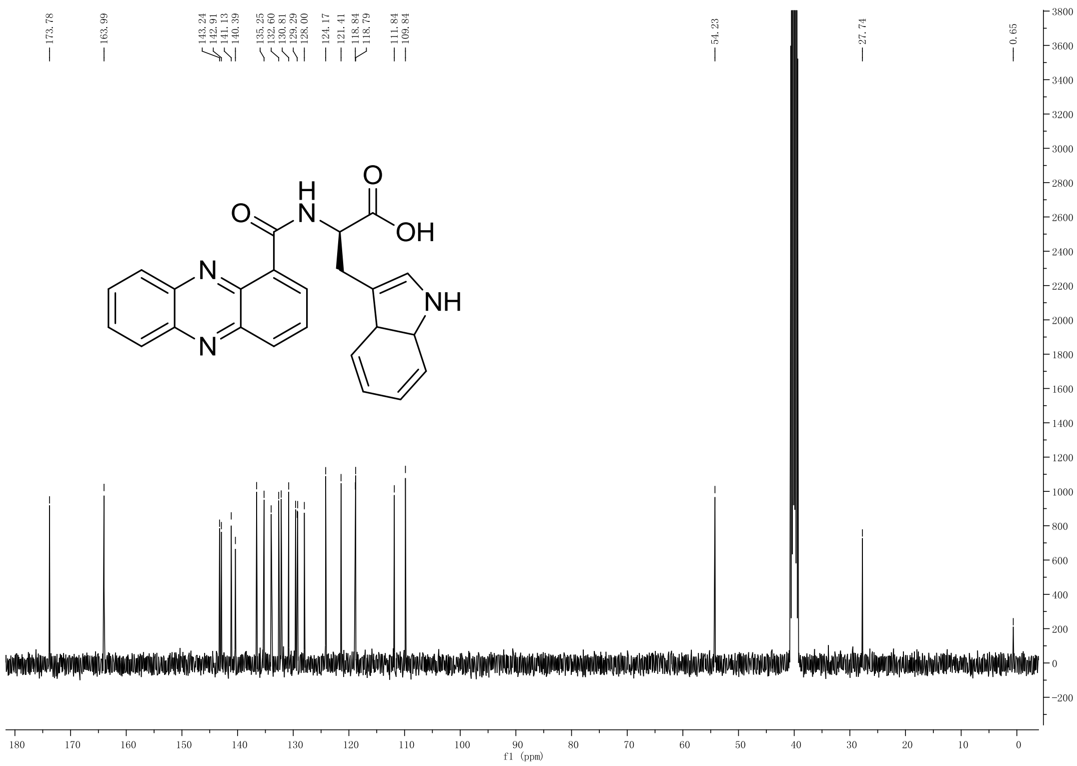

Figure S35. ^{13}C-NMR Spectrum of compound **4i**

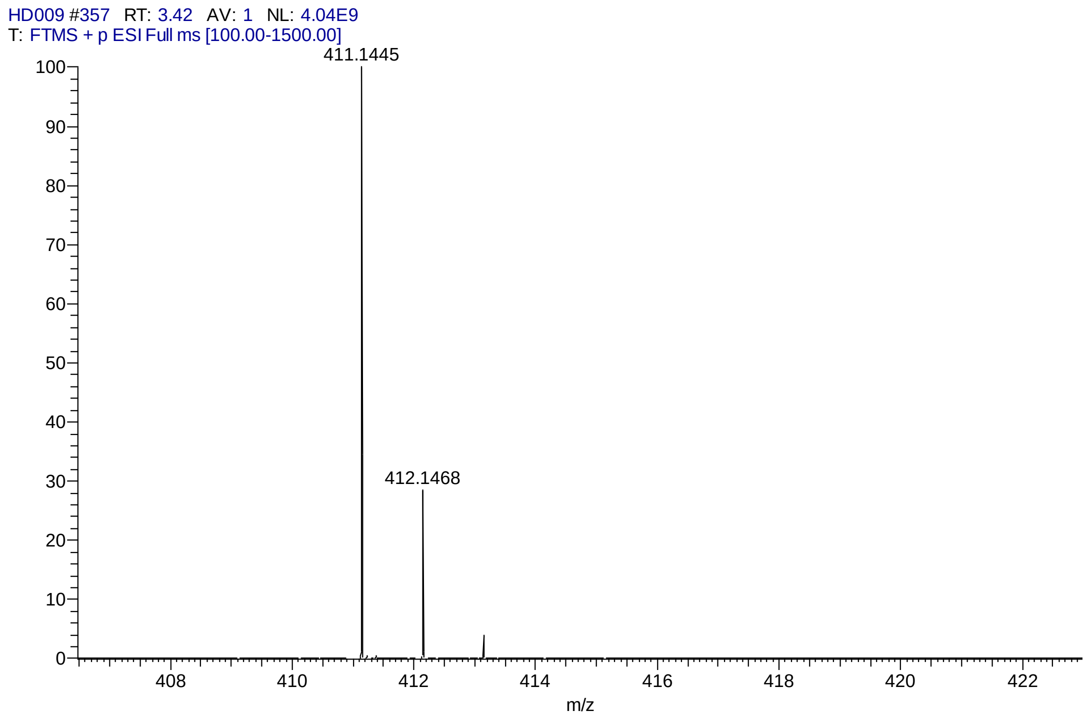

Figure S36. HRMS Spectrum of compound **4i**

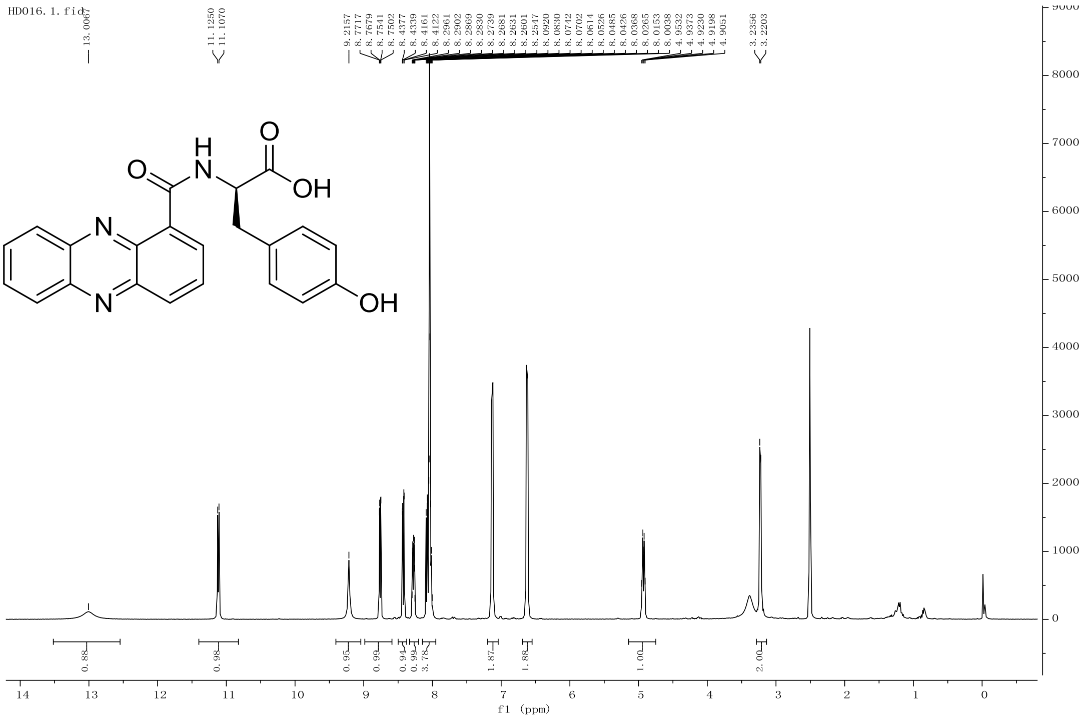

Figure S37. ^{1}H-NMR Spectrum of compound **4j**

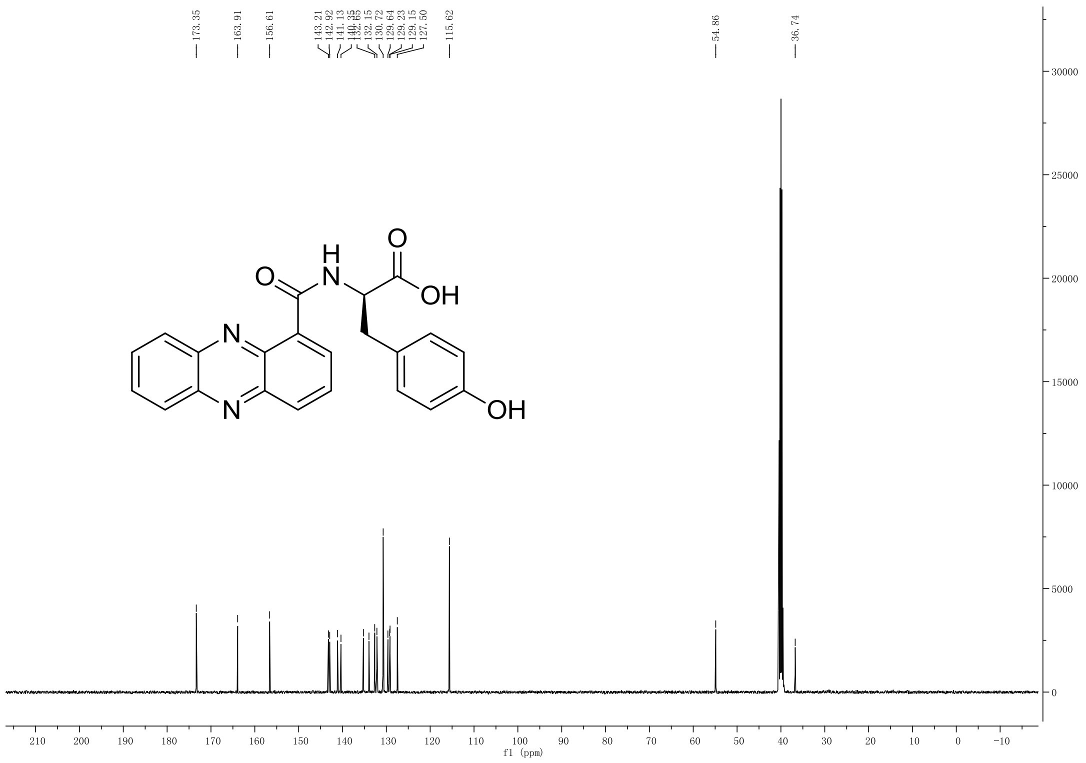

Figure S38. ^{13}C-NMR Spectrum of compound **4j**

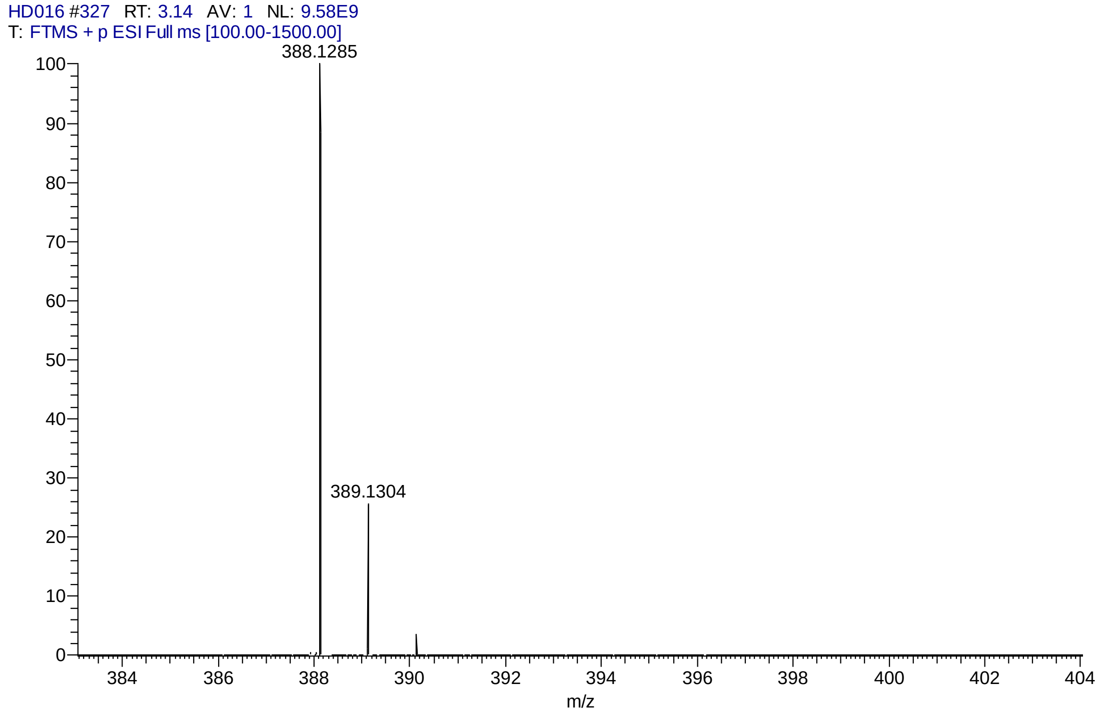

Figure S39. HRMS Spectrum of compound **4j**

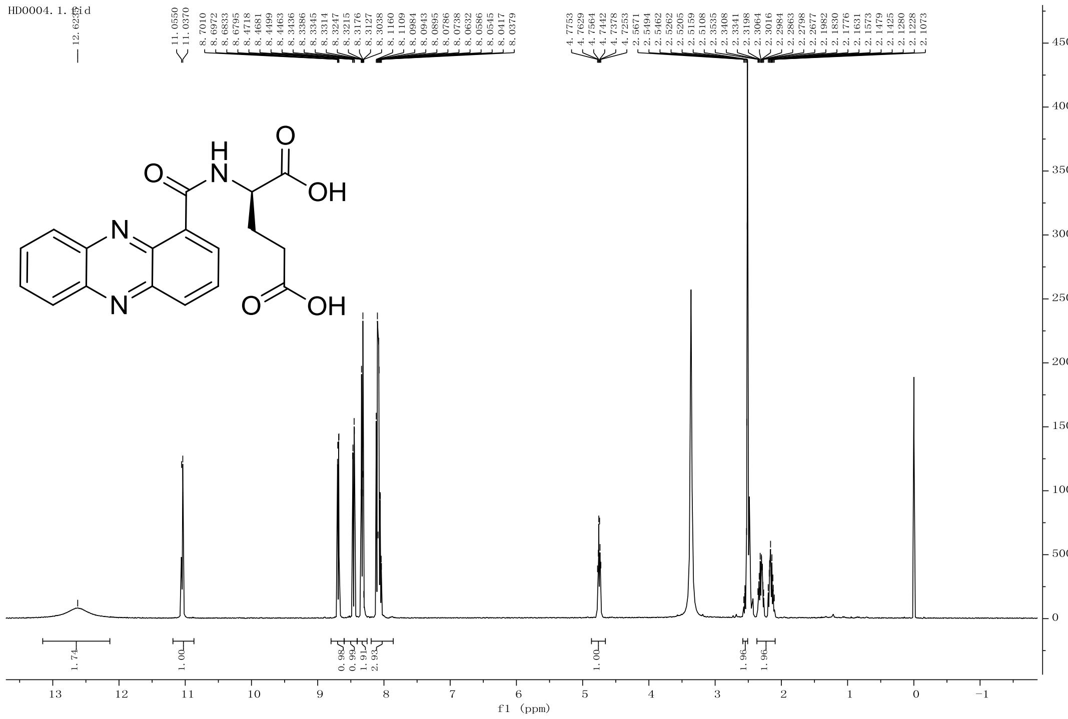

Figure S40. ^{1}H-NMR Spectrum of compound **4k**

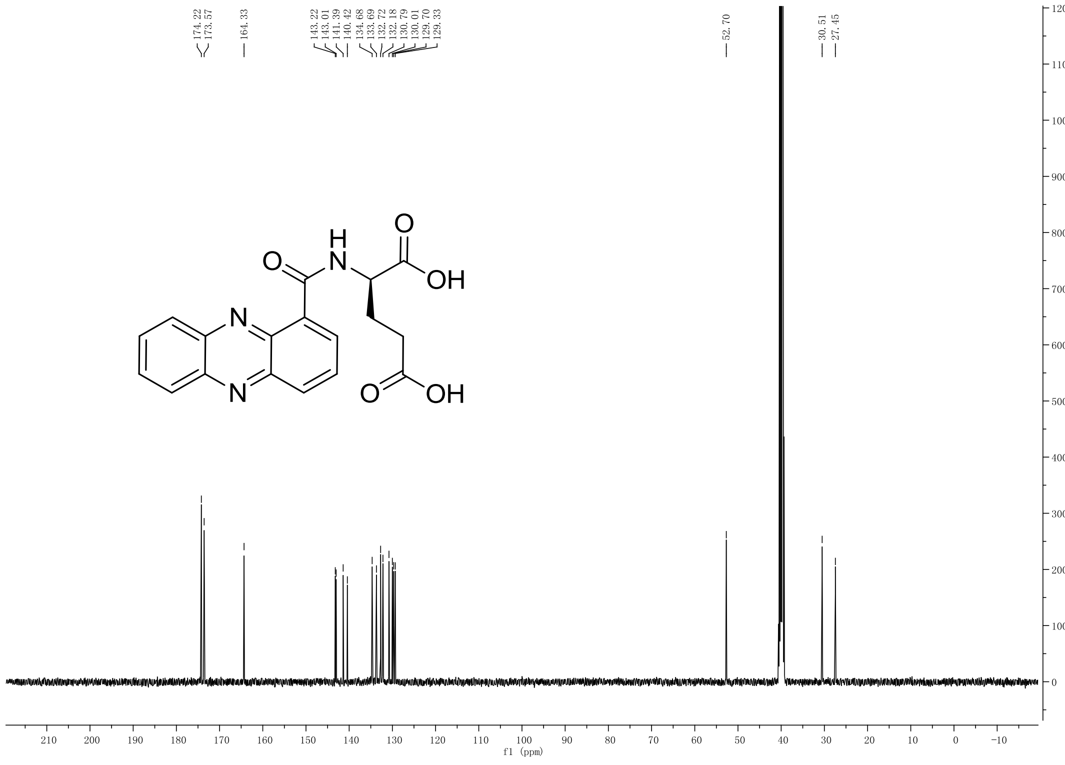

Figure S41. ^{13}C-NMR Spectrum of compound **4k**

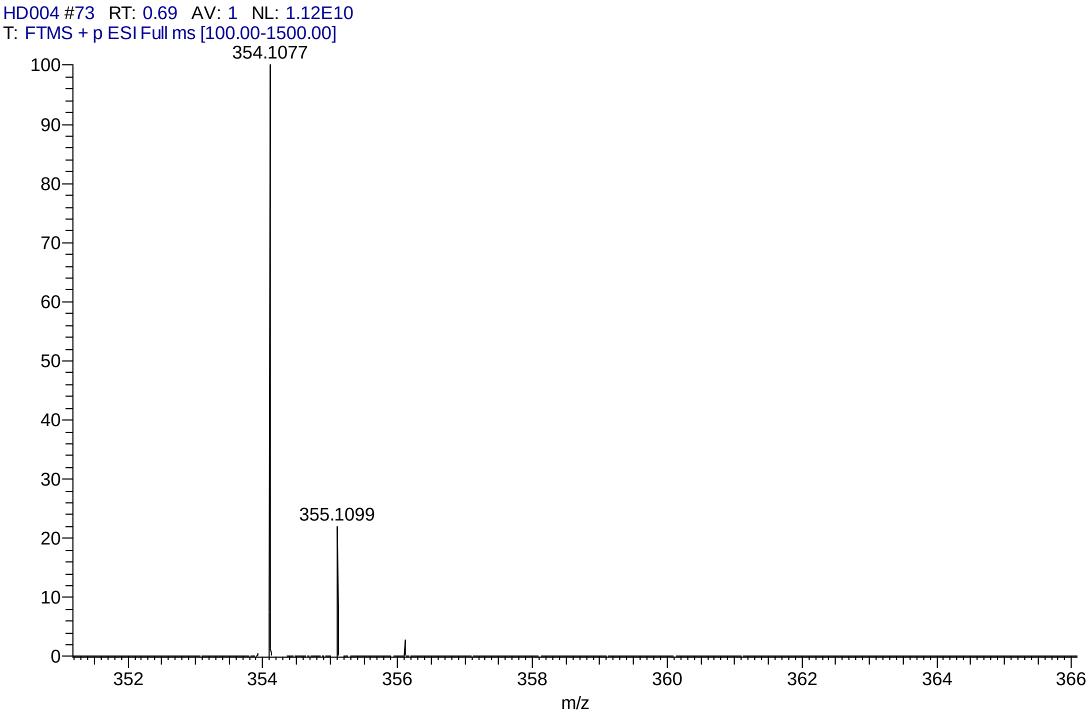

Figure S42. HRMS Spectrum of compound **4k**

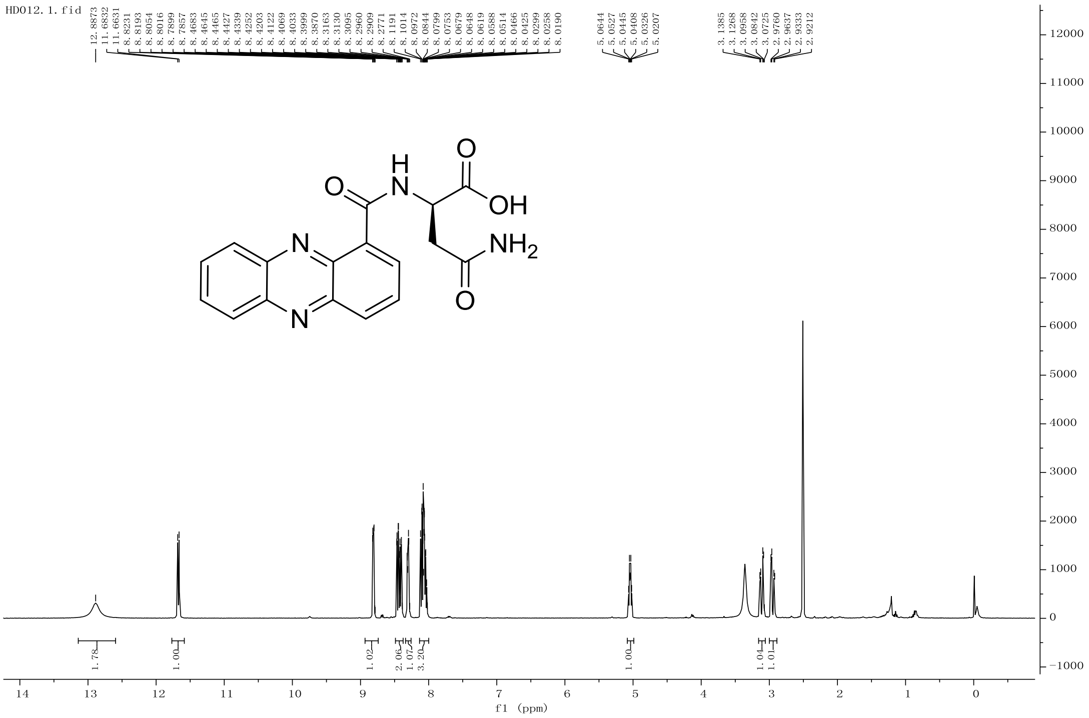

Figure S43. ^{1}H-NMR Spectrum of compound **4l**

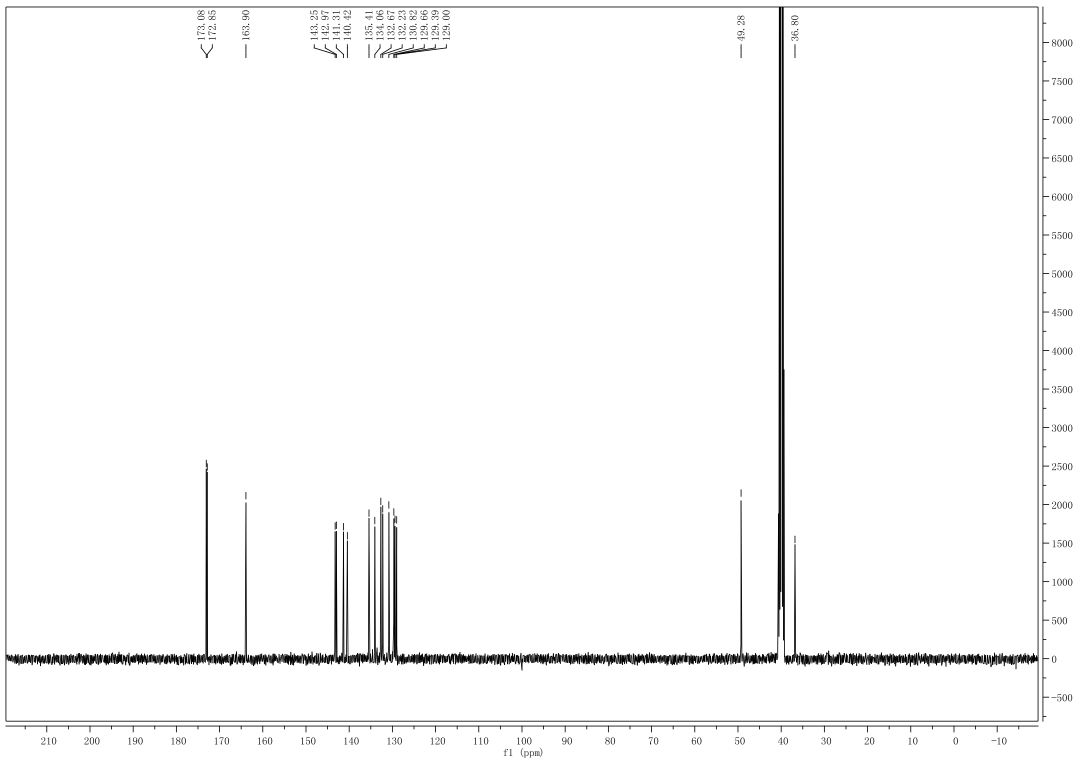

Figure S44. ^{13}C-NMR Spectrum of compound **4l**

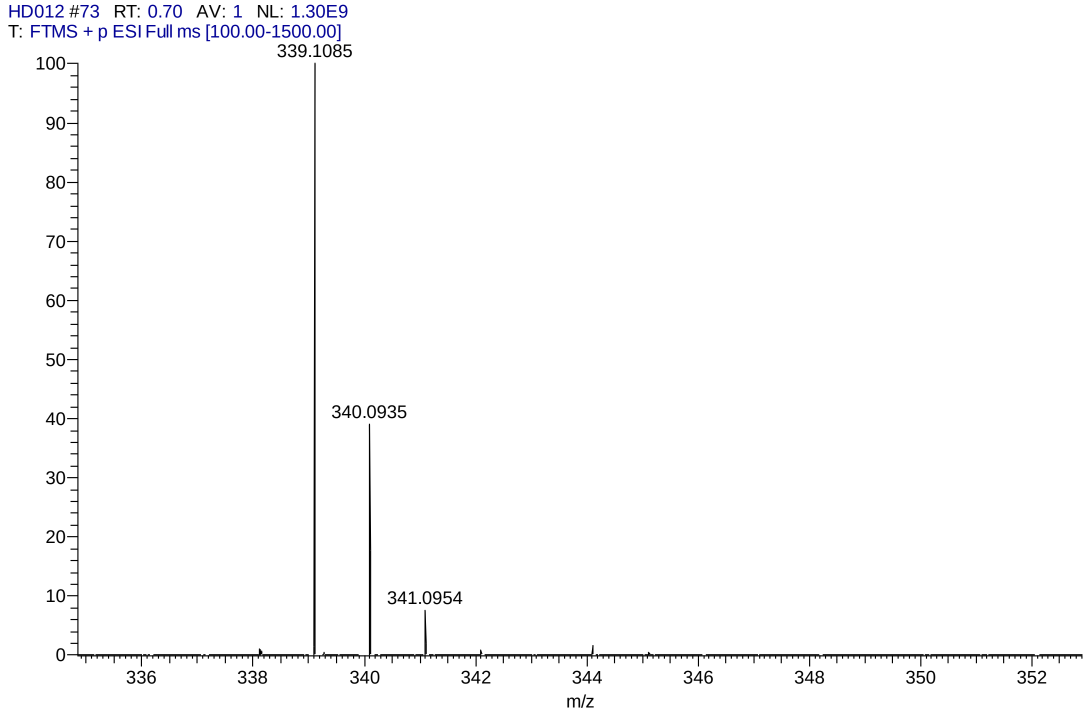

Figure S45. HRMS Spectrum of compound **4l**
